# Chamber Specific Gene Expression Landscape of the Zebrafish Heart

**DOI:** 10.1371/journal.pone.0147823

**Published:** 2016-01-27

**Authors:** Angom Ramcharan Singh, Ambily Sivadas, Ankit Sabharwal, Shamsudheen Karuthedath Vellarikal, Rijith Jayarajan, Ankit Verma, Shruti Kapoor, Adita Joshi, Vinod Scaria, Sridhar Sivasubbu

**Affiliations:** 1 Genomics and Molecular Medicine, CSIR Institute of Genomics and Integrative Biology, Mathura Road, Delhi 110025, India; 2 GN Ramachandran Knowledge Center for Genome Informatics, CSIR Institute of Genomics and Integrative Biology, Mathura Road, Delhi 110025, India; 3 Academy of Scientific and Innovative Research, CSIR-IGIB South Campus, Mathura Road, Delhi 110025, India; Mayo Clinic, UNITED STATES

## Abstract

The organization of structure and function of cardiac chambers in vertebrates is defined by chamber-specific distinct gene expression. This peculiarity and uniqueness of the genetic signatures demonstrates functional resolution attributed to the different chambers of the heart. Altered expression of the cardiac chamber genes can lead to individual chamber related dysfunctions and disease patho-physiologies. Information on transcriptional repertoire of cardiac compartments is important to understand the spectrum of chamber specific anomalies. We have carried out a genome wide transcriptome profiling study of the three cardiac chambers in the zebrafish heart using RNA sequencing. We have captured the gene expression patterns of 13,396 protein coding genes in the three cardiac chambers—atrium, ventricle and bulbus arteriosus. Of these, 7,260 known protein coding genes are highly expressed (≥10 FPKM) in the zebrafish heart. Thus, this study represents nearly an all-inclusive information on the zebrafish cardiac transcriptome. In this study, a total of 96 differentially expressed genes across the three cardiac chambers in zebrafish were identified. The atrium, ventricle and bulbus arteriosus displayed 20, 32 and 44 uniquely expressing genes respectively. We validated the expression of predicted chamber-restricted genes using independent semi-quantitative and qualitative experimental techniques. In addition, we identified 23 putative novel protein coding genes that are specifically restricted to the ventricle and not in the atrium or bulbus arteriosus. In our knowledge, these 23 novel genes have either not been investigated in detail or are sparsely studied. The transcriptome identified in this study includes 68 differentially expressing zebrafish cardiac chamber genes that have a human ortholog. We also carried out spatiotemporal gene expression profiling of the 96 differentially expressed genes throughout the three cardiac chambers in 11 developmental stages and 6 tissue types of zebrafish. We hypothesize that clustering the differentially expressed genes with both known and unknown functions will deliver detailed insights on fundamental gene networks that are important for the development and specification of the cardiac chambers. It is also postulated that this transcriptome atlas will help utilize zebrafish in a better way as a model for studying cardiac development and to explore functional role of gene networks in cardiac disease pathogenesis.

## Introduction

Zebrafish has been widely used as a vertebrate model to understand human cardiac development, function and many key aspects of cardiac disease manifestations [1]. The physiology of the zebrafish heart parallels that of the human heart in many aspects [2]. In both humans and zebrafish, the heart is a muscular structure having demarcated chambers with valves and performs the pumping function in a regular, rhythmic way to ensure uniform directional flow of oxygen-carrying blood. Several amputation and cryoinjury based models have been also developed in zebrafish to understand wound response and fundamentals of *in vivo* regeneration [3]. In both humans and zebrafish, a conserved network of cardiac patterning genes, transcriptional factors, cell adhesion molecules and signalling pathways operate as the early heart tube transforms into a chambered heart [2]. Zebrafish, is thus emerging as a clinically relevant model for studies related to genetic and pharmacological factors affecting heart function and repair. To harness the full potential of zebrafish models of cardiac disease and repair, a complete profiling of gene expression of the adult zebrafish cardiac tissue is required. Such information can facilitate translational advancements using cardiac disease modelling in zebrafish.

Expression of cardiac chamber genes is vital for normal development and function of the heart. A complete signature of cardiac gene expression can provide critical insights into normal chamber development and compartment specific function of the heart. Availability of a complete transcriptome atlas may help identify complete set of genes associated with a patho-physiology such as heart failure, which is otherwise an exigent task [4]. A number of high throughput methods have tried to identify gene signatures that are involved in cardiac development and disease etiology [4] [5]. High density oligonucleotide microarrays have been popularly used for understanding transcriptome profiles in the context of deciphering genetic networks involved in certain disease pathologies. Often, the microarray data is validated by quantitative real time PCR, which is another standard method for measuring gene expression levels [6]. Microarray based expression profiling to define the key proteins and genes involved in cardiac development and physiology has been performed for human fetal heart development [7], cardiac injury [8], lineage analysis of cardiac progenitors [9], atrial fibrillation [10], dilated cardiomyopathy [11], induced cardiac remodelling [12] and congenital heart defects [13]. Apart from describing the alterations in coding component of the genome, the perturbations to the non coding RNA in ischemia/reperfusion [14], atrial fibrillation [15], heart failure [16], fetal heart development [17] and metabolism related derangements affecting cardiac failure [18] have also been undertaken. However, the significance of information regarding the gene expression data available through such studies is partial. Often, the knowledge of crucial cell signalling pathways active in primary processes such as myocardial cell differentiation and the genetic regulation of cardiac specific gene expression is lacking [19]. Further, the genomic coverage provided by a microarray study is often restricted to known genes and lacks reproducibility when different platforms are used [20]. Thus, such data falls short of providing a detailed representation of the transcriptome landscape and genetic networks thus revealed do not fit perfectly with the documented disease pathways. One of the key reflections on this principal problem is that there is a lack of resources that expound gene expression profiles corresponding to basal physiology and normal development. The entire framework of cardiac gene expression is required as the standard for comparison in order to deduce the unknown genetic perturbations. Such a resource would ensure discovery of genetic and molecular networks that can be aligned flawlessly with known pathways and facilitate the discovery of novel partners pertinent to cardiac biology.

Deep RNA sequencing has emerged as a robust method that overcomes the caveats of microarray based gene expression profiling [21]. RNA sequencing does not require any prior knowledge of expressed gene sequence or primer design and can analyze whole RNA transcriptomes from any given tissue. RNA sequencing can detect completely unknown transcripts and novel genes [22], splice variants [23,24], changes in DNA sequence and can generate complete genomic coverage with detailed information on the transcriptional landscape. This approach has been widely adopted in a number of studies for cardiac transcriptome profiling to explore transcripts that are otherwise not identified using high density oligonucleotide arrays [25–27]. In the adult mammalian heart, the expression patterns of chamber-restricted protein coding genes have been characterized [19,28–33]. These studies highlighted the necessity for an inclusive understanding of genetic regulatory mechanisms that are involved in heart chamber specification.

We have employed an ultra-deep sequencing approach to identify the transcriptional profiles of the three heart chambers in adult zebrafish. We have captured the gene expression patterns of 13,396 genes in the three cardiac chambers—atrium, ventricle and bulbus arteriosus. Of this repertoire, a total of 7,260 known protein coding genes were found to show high expression of ≥10 FPKM (Fragments Per Kilobase of transcripts per Million mapped reads) in the zebrafish heart. The transcriptome data, thus consists of a near to complete set of all transcripts that are expressed in the zebarfish cardiac tissue. We identified 96 known protein coding genes that displayed chamber-restricted expression patterns. Using differential expression analysis, we have identified genes that are significantly restricted to a specific chamber and found that the atrium, ventricle and bulbus arteriosus had 20, 32 and 44 uniquely expressing genes respectively. Using a *de novo* transcript assembly approach, we identified 23 putative novel protein coding genes that were restricted only to the zebrafish ventricle. Out of the 96 chamber specific differentially expressed genes, 68 genes are known to have human orthologs, a few of which are documented to have cardiac disease associations. We believe that this transcriptome atlas together with the information from other high throughput approaches will be advantageous in creating a cardiac disease centric interactome. This comprehensive transcriptome can function as a reference catalogue for interpreting gene expression while modelling cardiac disease conditions in zebrafish.

## Materials and Methods

### Ethics Statement

All the zebrafish related experiments were performed strictly by following the guidance and procedures prescribed by the CSIR-Institute of Genomics and Integrative Biology (CSIR-IGIB), Delhi, India. Wild-type ASWT [34] fish housed at CSIR-IGIB were utilised for this study. The procedure adopted in the study has been approved by the Institutional Animal Ethics Committee (IAEC) of the CSIR- IGIB, India. Utmost care was taken to diminish any kind of suffering to the animals.

### Dissection of adult zebrafish heart and RNA isolation

Adult wild-type ASWT zebrafish were housed at fish facility, CSIR-IGIB, following the standard practices [35]. Tissue isolation was performed by anaesthetizing six month-old wild-type ASWT zebrafish (15 in number) by treatment with tricaine (Sigma, USA) and hearts were dissected out under a dissecting microscope (Axioscope 40 microscope, Carl Zeiss, Germany). The three chambers viz. atrium (A), ventricle (V) and bulbus arteriosus (BA) were carefully separated from 15 animals. Firstly, the atrium was removed from the heart, followed by ventricle and BA by using an iris scissors (Sigma, USA). Extreme precaution was taken to prevent contamination and to acquire pure samples for each chamber type. The individual cardiac chambers dissected from 15 animals were pooled to constitute one sample each for atrium, ventricle and bulbus arteriosus respectively. Total RNA was isolated by employing homogenization by Pellet pestles cordless motor (Z359971, Sigma) in trizol (Invitrogen, USA) followed by purification using a Qiagen RNeasy column (Qiagen) as per manufacturer’s protocol. RNA quality was assessed as previously described [36].

### Deep sequencing and data generation

Approximately, 1 μg of total RNA was used from each chamber of the wild type adult zebrafish heart to prepare libraries for sequencing by using Truseq stranded RNA library preparation kit according to the manufacturer’s supplied protocol (Illumina Inc. USA). Ribosomal RNA (rRNA) in each sample was depleted as per manufacturer's instructions (Illumina Inc. USA) followed by fragmentation. Complementary DNAs (cDNAs) were prepared after rRNA depletion. Further, sequencing adapters were ligated and the enriched libraries were subjected to 101x 2 paired end sequencing using Hiseq 2500 platform following regular protocol (Illumina Inc. USA). The FASTQ sequencing reads obtained from the three chamber tissues were adapter-trimmed along with a quality cut-off of Q20 and a minimum length cut-off of 30 bases using Trimmomatic [37]. The reads were aligned to the latest available zebrafish genome assembly (Zv9) using the popular splice-aware aligner, STAR [38].

### Identification of putative novel cardiac chamber specific genes

In order to discover novel transcripts in the cardiac chambers, we used the STAR-aligned reads to perform a *de novo* transcriptome assembly using Cufflinks [39]. The individual chamber transcriptomes were then merged using Cuffmerge to obtain a comprehensive transcriptome assembly representing all the three cardiac chambers. We then employed an expression level cut-off of 10 FPKM (Fragments Per Kilobase of transcript per Million mapped reads) to select transcripts to be further processed for the identification of novel protein coding transcripts. Here, we define transcripts that are longer than 200 nucleotides (nt) and have protein coding open reading frames (ORF) longer than 30 amino acids, as putative novel protein coding genes. Firstly, known protein coding genes were excluded by overlapping with the latest zebrafish RefSeq and Ensembl (v79) gene catalogue. The transcript sequences were downloaded from UCSC Table browser and filtered for transcripts longer than 200 nt. Getorf program from the EMBOSS suite [40] was used to predict ORFs within the transcript sequences. We then employed two tools, Coding Potential Calculator (CPC) [41] and PhyloCSF [42] to assess protein coding potential of the transcripts with longest ORF more than 30 amino acids using threshold scores of CPC > 1 and PhyloCSF > 100 and generated the final catalogue of putative novel protein coding transcripts. In order to annotate the transcripts with known protein domains, we employed HMMER3 [43] to query the predicted ORFs against Pfam protein database [44] with default parameters and a significant Pfam hit (E-value < 0.001).

### Differential expression analysis

We used Cuffdiff [39] to perform differential expression analysis for the known (RefSeq gene catalogue) as well as putative novel protein coding genes identified in this study. We used an expression fold-change threshold of at least 10 and an expression level (FPKM score) for the enriched gene to be at least 5 to obtain the final list of genes that were exclusive to cardiac chambers. To pick up the expression pattern of the cardiac chamber-restricted genes across developmental time points, we used Cuffdiff to compare their expression levels across 11 developmental stages (2–4 cell, 1000 cell, dome, shield, bud, 1 day post fertilization (dpf), 2dpf, 3dpf, 5dpf, 14dpf and adult) [45,46], Zebrafish transcriptome sequencing project [PRJEB1986] and 6 adult tissue types (ovary, blood, brain, lungs, liver and kidney) using publicly available datasets. We then used R [R Development Core Team] to generate heat maps to represent the expression patterns. Additionally, KEGG pathway enrichment analysis for all cardiac-restricted Refseq protein-coding genes that showed two-fold enrichment with a higher FPKM > = 5 was carried out using WebGestalt [47].

### Ribosome profiling of the chamber-restricted transcriptome

Ribo-seq reads from eight zebrafish developmental stages (2–4 cells, 256 cells, 1000 cells, dome, shield, bud, 28 hpf and 5 dpf) [48] were downloaded from NCBI SRA. Adapter trimming [49] was performed using fastx-clipper, a part of FASTX- Toolkit. Next, we removed the reads mapping to rRNAs after aligning the trimmed reads to zebrafish rRNA sequences downloaded from SILVA database [50] using Bowtie2 [51]. About 66% reads were discarded in this step. We then isolated 235 million (M) high-quality reads by placing a read length filter of 27–32 nt as described [48]. These high quality reads were then mapped using Tophat2 [52] to the merged *de novo* transcriptome assembly generated in this study and Zv9 reference genome, providing 90.3% alignment. We further downloaded RNA-Seq reads for 8 zebrafish developmental stages (2–4 cells, 1000 cells, dome, shield, bud, 28 hpf, 2dpf and 5 dpf) [45] from NCBI SRA. Following pre-processing using Trimmomatic [37] and SolexaQA [53], the reads were mapped to Zv9 reference genome using Tophat2 [52] obtaining a mapping percentage of 97%. To calculate Translation Efficiency Score (TES) for the putative novel protein-coding genes, we first used BED Tools [54] to obtain Ribo-seq and RNA-seq read counts overlapping each novel protein-coding feature. We then formulated TES for individual anticipated novel protein-coding gene as the ratio of its Ribo-seq read count to RNA-Seq mapped read count.

### Orthologs, mutants and human disease genes

Mutant phenotypes and human orthologs for the zebrafish cardiac genes were retrieved from Zebrafish model organism database (ZFIN: Zebrafish Information Network) [55]. Disease annotations for the human orthologous genes were obtained from OMIM database [56]. We integrated this information to further annotate the known cardiac chamber-restricted protein coding genes identified as a part of this study.

### Quantitative Real-Time Reverse Transcriptase–Polymerase Chain Reaction

RNA was isolated from the three chambers, atrium (A), ventricle (V) and bulbus arteriosus (BA) of the adult zebrafish heart using RNeasy kit (Qiagen) as per the instructions provided by the manufacturer. 1μg of total RNA and Superscript II (Invitrogen, USA) was used for first strand cDNA synthesis. Quantitative Real Time Polymerase Chain Reaction [6] (qRT-PCR) was performed and Sybr Green mix (Roche, Germany) was added to the PCR reaction for detection in Light cycler LC 480 (Roche). Primers for nine representative differentially expressed genes were designed (S1 Table) and qPCR was carried out in 96-well qPCR plates (Roche Diagnostics Corp). Three RNA samples (one each for A, V, BA) were analyzed in triplicates. Amplification was performed in triplicates at 58°C for 2 minutes (min) and 95°C for 5 min followed by 40 cycles of 95°C for 20 seconds (sec), 58°C for 20 sec and 72°C for 20 sec. Reactions without template and/or enzyme were used as negative controls. Beta-actin gene was used as an internal control. Fold differences were calculated by normalising the CT values obtained for each of the gene against chamber-restricted marker genes. We selected *myosin*, *heavy chain 6*, *cardiac muscle*, *alpha* (*myh6*) and *ventricular myosin heavy chain* (*vmhc*) as chamber-restricted control genes for atrium and ventricle respectively [57,58].

### Whole mount In Situ hybridization (WISH)

Heart tissues fixed in para formaldehyde were processed for *in situ* hybridization as per published protocols [59]. The chamber-restricted protein coding gene sequences were amplified from cDNA by PCR using gene specific primers (S1 Table) and cloned into Topo TA vector (Invitrogen, USA). The clones were linearized with Spel enzyme (NEB, USA) and digoxygenin (DIG) labeled *in situ* probes were synthesised by *in vitro* transcription with SP6 or T7 polymerases using DIG RNA labelling kit (Roche, Germany).

## Results

### Chamber specific RNA isolation

The sample pool used for RNA sequencing in the present analysis included cardiac tissue dissected from the three chambers (A, V and BA) and pooled from 15 animals as described in the Methods section. The heart anatomy and representative images of the whole heart and the three dissected chambers are shown (S1 Fig). RNA was isolated and checked for quality (S2 Fig). The RNA samples were further processed for rRNA depletion and cDNA was prepared from each of the samples independently. To validate the accuracy of dissection, and purity of the dissected chamber samples, we checked the expression of previously known chamber-restricted gene markers using quantitative real-time PCR (qRT-PCR). We used two genes, *myh6* and *vmhc*,that have previously been described to show chamber-restricted expression respectively in atrium and ventricle of the zebrafish heart (S3 Fig). The qRT-PCR data confirmed the accuracy of dissection and purity of the RNA samples from different chambers of the zebrafish heart.

### RNA sequence data generation and reference mapping

**Fig 1:**
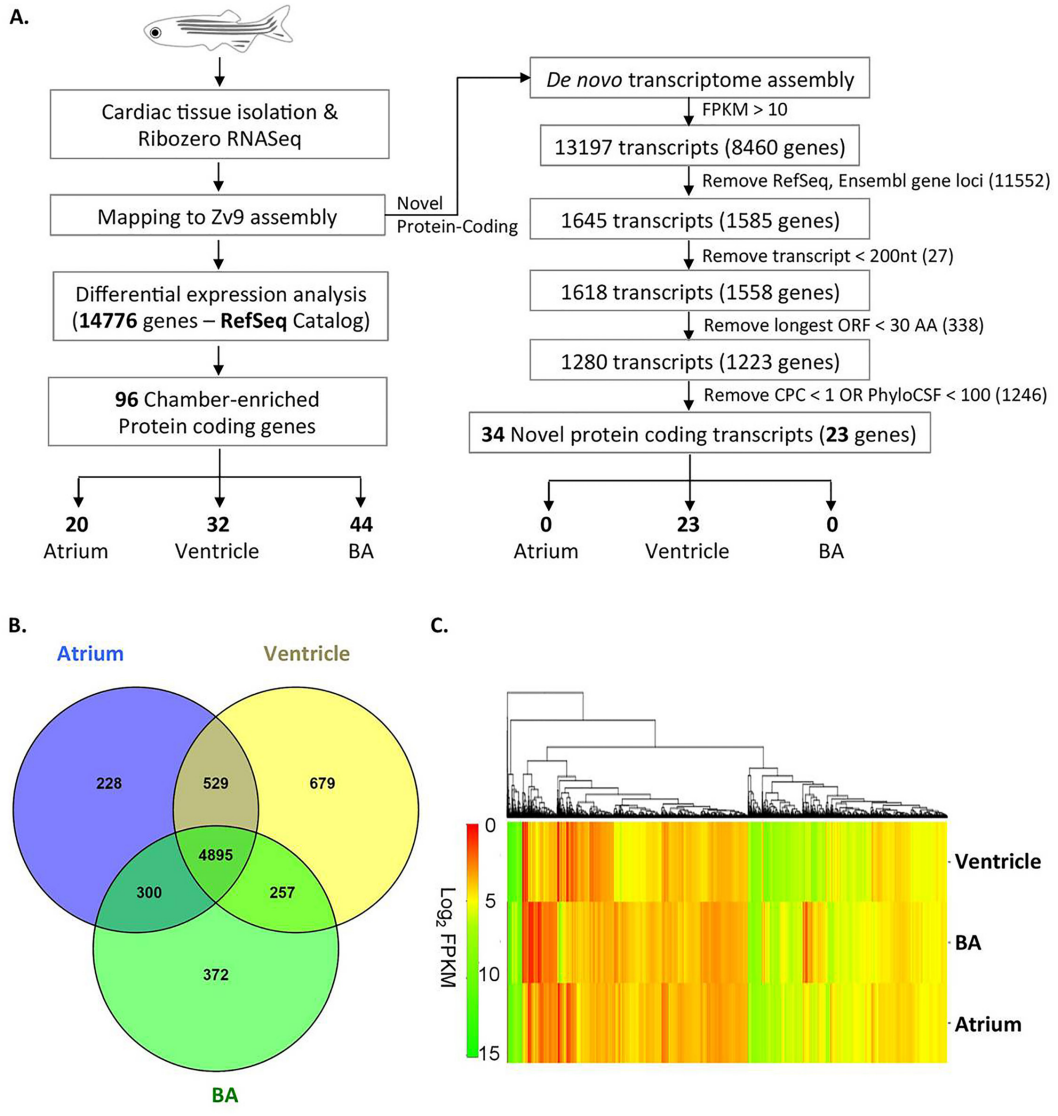
Data workflow and analysis summary. An overview of experimental and data analysis workflow adopted in the study for identification of known and putative novel cardiac chamber-restricted protein coding genes. (B) Venn diagram representing the total number of known protein coding genes (7,260) identified with expression level > = 10 FPKM across the three cardiac chambers. (C) A heat map representing expression pattern of 7,260 RefSeq protein coding genes with expression level > = 10 FPKM in the three cardiac chambers. The colour key represents transcripts in the range of 0 for transcripts with least expression to 15 for transcripts with maximum expression.

Ribosomal RNA depleted, strand specific and 101 base pair (bp) paired end raw sequence reads were generated using Hiseq 2500 by employing sequence-by-synthesis method (Illumina Inc., USA). Approximately, 735 million raw paired-end sequence reads of 101 bp were obtained from the three chamber libraries. The sequence reads were aligned to the zebrafish reference genome (Ensembl Zv9 build; hereafter called as Zv9) after eliminating low quality bases and adapter contamination. Approximately, 600 million (84%) sequencing reads were successfully mapped back to the reference genome using STAR (Table 1). These mapped reads were used for further analysis. The approach used for data processing and analysis is summarized (Fig 1A). The raw transcriptome sequences have been submitted to SRA with accession numbers SRX1153632 (Ventricle), SRX1153633 (Atrium) and SRX1153634 (Bulbus arteriosus).

**Table 1:** Summary of the RNA sequencing data generation and alignment. Total number of sequence reads obtained from the three cardiac chambers using RNA sequencing is described. Mapped reads refer to and include all transcripts that aligned back to the zebrafish reference genome (Zv9). The total number of uniquely expressing known protein coding genes as well as *de novo* transcripts are mentioned.

	Atrium	Ventricle	Bulbus arteriosus	Total (Unique)
Total reads (in millions)	210	255	270	735
Post-trimming (in millions)	198	249	263	710
Mapped reads (in millions)	162 (82%)	226 (91%)	212 (80%)	600 (84%)
Known Protein Coding Genes with expression ≥1 FPKM, RefSeq Catalog	13,105	10,567	12,404	13,396
Known Protein Coding Genes with expression ≥10 FPKM, RefSeq Catalog	5,951	6,359	5,823	7,260
*De novo* assembled Transcripts (Genes) with expression ≥10 FPKM	497 (352)	12,926 (8,388)	669 (442)	13,197 (8,460)

### Cardiac transcriptome identification in adult zebrafish

The aligned reads that mapped to the zebrafish genome were used to estimate the expression of known protein coding genes in adult zebrafish heart chambers. Briefly, we used Cuffdiff to compute normalized expression levels and perform differential expression analysis for the annotated RefSeq genes (14,776 genes) in the three cardiac chambers (Fig 1A). Further, we selected the genes with FPKM > = 1 and identified 13,396 protein coding genes (Table 1). We analyzed the distribution as well as the expression pattern of highly expressed cardiac genes (FPKM > = 10; 7,260 genes) within the cardiac chambers (Table 1; Fig 1B and 1c) and interestingly, observed a remarkable overlap of 529 genes between atrium and ventricle, 300 genes between atrium and bulbus arteriosus and 257 between ventricle and bulbus arteriosus. A subset of genes were found to be highly expressed (FPKM > = 10) specific to individual chambers (679 genes in ventricle, 228 genes in atrium and 372 in bulbus arteriosus). For differential expression analysis, genes with a higher expression (higher FPKM score) in a particular chamber relative to the other two chambers were chosen. We identified a total of 96 protein coding genes (Fig 1A) showing at least 10 fold enrichment across the three cardiac chambers.

### Differential gene expression across the chambers

Our analysis identified a total of 5,951 genes expressing in the atrium and 5,823 genes in the bulbus arteriosus. Of the three chambers, maximum number of genes was identified in the ventricle i.e., 6,359 (The complete list of the genes expressing in the three chambers is available online as additional data at http://zebrafish.igib.in/zebrafish-heart-transcriptome). The RNA-seq data analysis revealed that the atrium and ventricle have distinct gene expression profiles. Cuffdiff analysis at a false detection rate of 0.001 identified 20 out of 5,951 genes with a restricted expression in the atrium and 32 out of 6,359 genes with expression constrained to the ventricle. Bulbus arteriosus, showed the maximum number of unique genes, about 44 that showed restricted expression in this chamber (Fig 1A).

**Fig 2:**
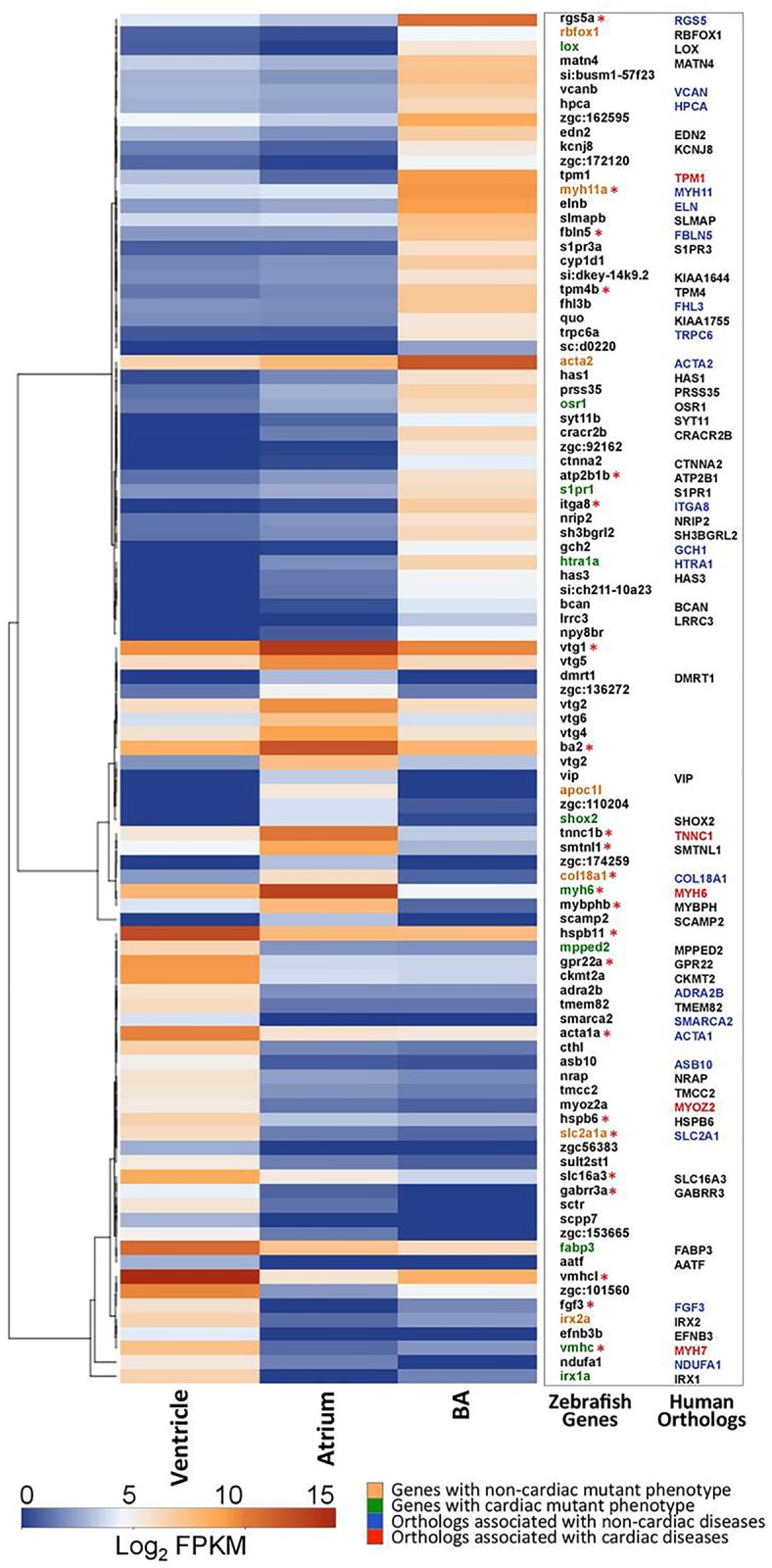
Differential expression of genes in the three cardiac chambers. The heat map represents expression profile of 96 cardiac chamber-restricted genes. The first column in the adjoining table lists the corresponding zebrafish gene names which are colour coded to highlight those with mutant phenotypes. Genes marked with green colour signify those which have been identified with heart related mutant phenotypes. The second column lists the human orthologs known for these genes. Genes marked with asterisks (*) represent those mentioned in the text (Results & Discussion). Colour coding for the human orthologs highlights those which show disease associations. Orthologs marked with red colour are associated with cardiac disorders. The colour key represents transcripts in the range of 0 for transcripts with least expression to 15 for transcripts with maximum expression.

Our analysis showed 5,951 genes to be expressing in the atrium, of which the genes with highest expression include *myosin binding protein Hb* (*mybphb*), *troponin C type 1b*, *slow* (*tnnc1b*), *smoothelin like 1* (*smtnl1*) and *collagen type XVIII*, *alpha 1* (*col18a1*) (Fig 2). The zebrafish *tnnc1* functions in striated muscle contraction and when mutated, results in contractile defects and unusual chamber morphology [60]. The human *TNNC1* (OMIM: 191040) mutations are reported to cause hypertrophic cardiomyopathy 13 [61] and dilated cardiomyopathy DCM [62]. *SMTNL1* (OMIM: 613664), the human ortholog of *smoothelin like 1* acts in skeletal and smooth muscle fibres to regulate contraction-relaxation processes [63]. *COL18A1* (OMIM: 120328) mutations affect non-cardiac tissues and have not been implicated in cardiac disease [64]. *Myosin heavy chain 6* (*myh6*), the earliest marker that expresses in the zebrafish atrium, was found to be highly expressed in our transcriptome data as well [65]. *Myh6* is one of the important vertebrate sarcomeric genes and its role in contractile function in zebrafish was uncovered via the *wea* mutant (*weak atrium*) [57]. *MYH6* (OMIM: 160710) mutations in humans are known to cause familial hypertrophic cardiomyopathy and atrial septal defects [66]. *MYH6* gene mutations also lead to sick sinus syndrome in humans [67]. Apart from the contractile genes, a class of lipid transport genes was found to be significantly restricted to the atrium. These include, *vitellogenin 1*, *2*, *4* and *5* (*vtg1*, *vtg2*, *vtg4* and *vtg5*). This subset of genes does not have any known human orthologs and no mutations in zebrafish have been reported so far.

The transcript repertoire of the zebrafish ventricle tissue showed differences from that of the atrium and bulbus arteriosus (Fig 2). As per our analysis, membrane receptor genes such as *G protein-coupled receptor 22a* (*gpr22a*), *gamma-aminobutyric acid* (*GABA*) *receptor* and *rho 3a* (*gabrr3a*) showed a ventricle- restricted expression. The gene, *actin*, *alpha 1a*, *skeletal muscle* (*acta1a*), that encodes for skeletal muscle alpha-actin in zebrafish, was found to be highly expressed in the ventricle. *ACTA1* (OMIM: 102610) is localized to the thin filament of the sarcomere, is involved in muscle contraction and when mutated, leads to severe forms of myopathy [68]. The zebrafish ortholog of *CKMT* (OMIM: 123295), *creatine kinase*, *mitochondrial 2a* (*sarcomeric*) (*ckmt2a*), an isoenzyme that plays a crucial role in energy metabolism, and is expressed particularly in tissues with high energy requirements [69], was also observed to display ventricle- restricted expression. A few heat shock proteins such as *heat shock protein*, *alpha-crystallin*-*related*, *b6* (*hspb6)* [70] or *HSPB6* (OMIM: 610695), that participate in smooth muscle relaxation [71] and *heat shock protein*, *alpha-crystallin-related*, *b11* (*hspb11*) were also seen to be restricted to the ventricle.

As expected, *ventricular myosin heavy chain* (*vmhc*), an early marker for the zebrafish ventricle [57] and *ventricular myosin heavy chain-like* (*vmhcl*) [72] transcripts were found to be highly expressed in the ventricle. Alterations in human *MYH7* (OMIM: 160760) are associated with cardiomyopathies [73]. In addition, *fatty acid binding protein 3*, *muscle and heart* (*fabp3*), *fibroblast growth factor 3* (*fgf3*), *solute carrier family 2 facilitated glucose transporter member 1a* (*slc2a1a*), and *solute carrier family 16 monocarboxylate transporter*, *member 3* (*slc16a3*) were also found to show restricted expression in the ventricle.

The bulbus arteriosus expressed a unique transcript signature, mostly dominated by contractile proteins (Fig 2) such as *actin*, *alpha 2*, *smooth muscle*, *aorta* (*acta2*); *myosin*, *heavy chain 11a*, *smooth muscle* (*myh11a*); *integrin*, *alpha 8* (*itg8*); *tropomyosin 1*(*tpm1*); ATP binding gene, *ATPase*, *Ca*^*2+*^ *transporting*, *plasma membrane 1b* (*atp2b1b*) and *tropomyosin 4b* (*tpm4b*). Other highly expressed genes in the zebrafish bulbus arteriosus included *fibulin 5* (fbln5), *elastin b* (*elnb*) and *regulator of G-protein signalling 5a* (*rgs5a*) [74].

### Validation of differentially expressed known protein coding genes in cardiac chambers using qRT-PCR and whole mount *in situ* hybridization

**Fig 3:**
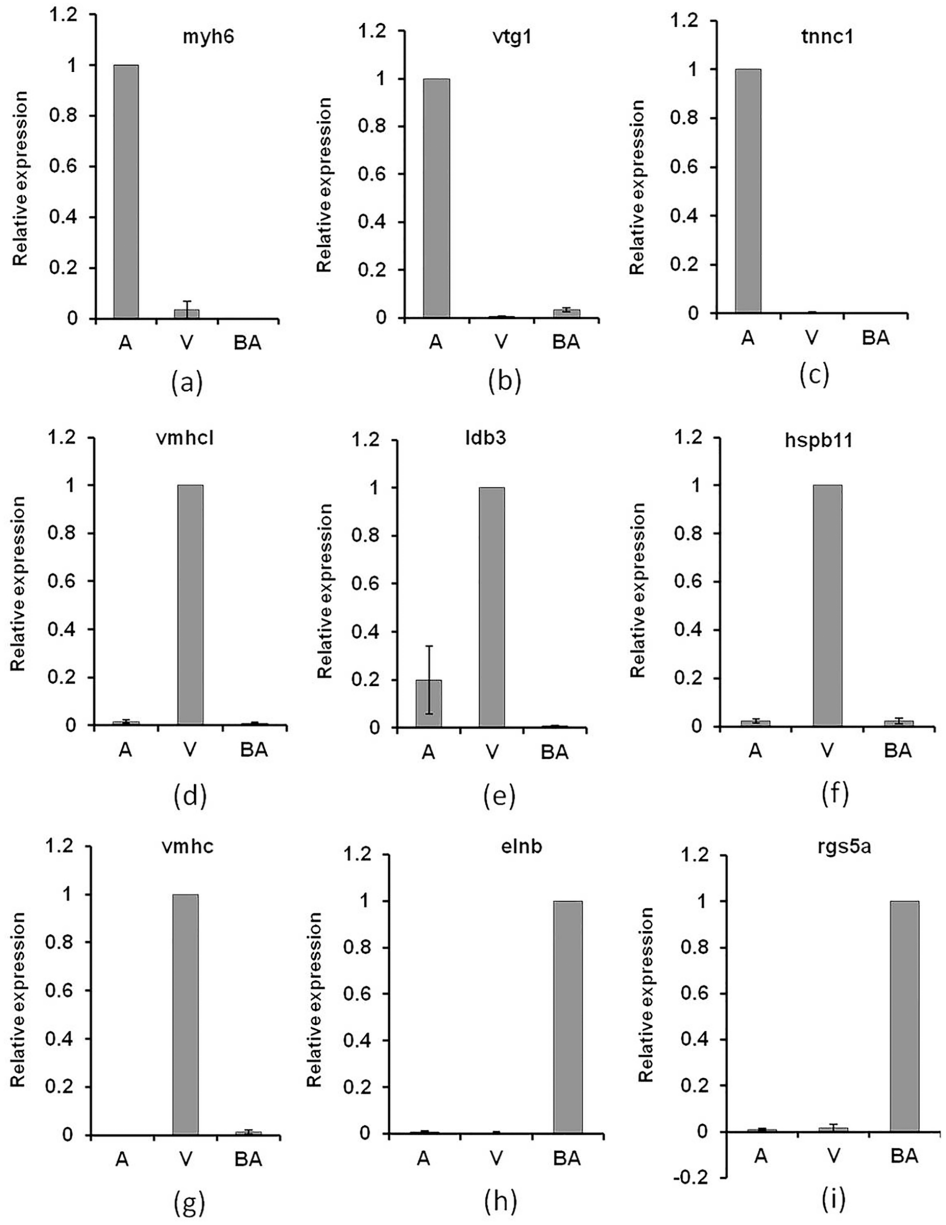
Quantitative Real time PCR based validation of chamber-restricted candidate genes. Atrium-restricted genes (a), (b) and (c). Ventricle-restricted genes (d), (e), (f) and (g). Bulbus arteriosus-restricted genes (h) and (i). See text for detailed information.

A subset of minimum two representative known protein coding genes per chamber was validated and confirmed by qRT-PCR. The atrium- restricted genes assayed, included *vitellogenin 1*(*vtg1*)*; myosin*, *heavy chain 6*, *cardiac muscle*, *alpha* (*myh6*) *and troponin c type 1*, *slow* (*tnnc1*). The ventricle-restricted genes assayed, included *ventricular myosin heavy chain* (*vmhc*), *ventricular myosin heavy chain like* (*vmhcl*), *LIM domain binding 3b* (*ldb3*), *heat shock protein*, *alpha-crystallin-related*, *b11* (*hspb11*) and the bulbus arteriosus-restricted genes assayed included *rgs5a and elastin b* (*elnb*). Overall, we observed that the differential expression pattern of these genes across chambers as predicted by the RNA sequencing data was evident when their relative expression were measured semi quantitatively using qRT-PCR (Fig 3).

We additionally carried out whole mount *in situ* hybridization to validate the chamber- restricted expression of differentially expressed protein coding genes. For ventricle-restricted gene, we assayed *irx1a*, that showed a 90 fold enrichment in the ventricle (FPKM = 90.75) as compared to atrium (FPKM = 0.00027) and observed an expression that was restricted to the ventricular chamber (S5A Fig). *ldb3a*, another ventricle-restricted gene, was found to have threefold higher expression in the ventricle (FPKM = 307.8) when compared to atrium (FPKM = 109.82). We observed that *ldb3a*, displayed a expression constrained to the ventricle apart from a moderate expression observed in atrium (S5B Fig). Similarly, *vtg2* displayed a 12 fold enrichment in the atrium (FPKM = 711.32) when compared to ventricle (FPKM = 62.94) and bulbus arteriosus (FPKM = 64.37). We observed an enriched *in situ* signal for *vtg2* in the atrial chamber (S5C Fig). For bulbus arteriosus-restricted gene, we selected *rgs5a* (FPKM = 1522.06) that displayed a 95 fold enrichment in the bulbus arteriosus when compared to ventricle (FPKM = 16.9) and 165 fold enrichment when compared to atrium (FPKM = 9.19). We observed that expression of *rgs5a* was strictly confined to the bulbus arteriosus (S5D Fig). Thus, we were able to verify the chamber-restricted differential expression of protein coding genes.

### Developmental expression profiles of cardiac chamber-restricted protein coding genes

**Fig 4:**
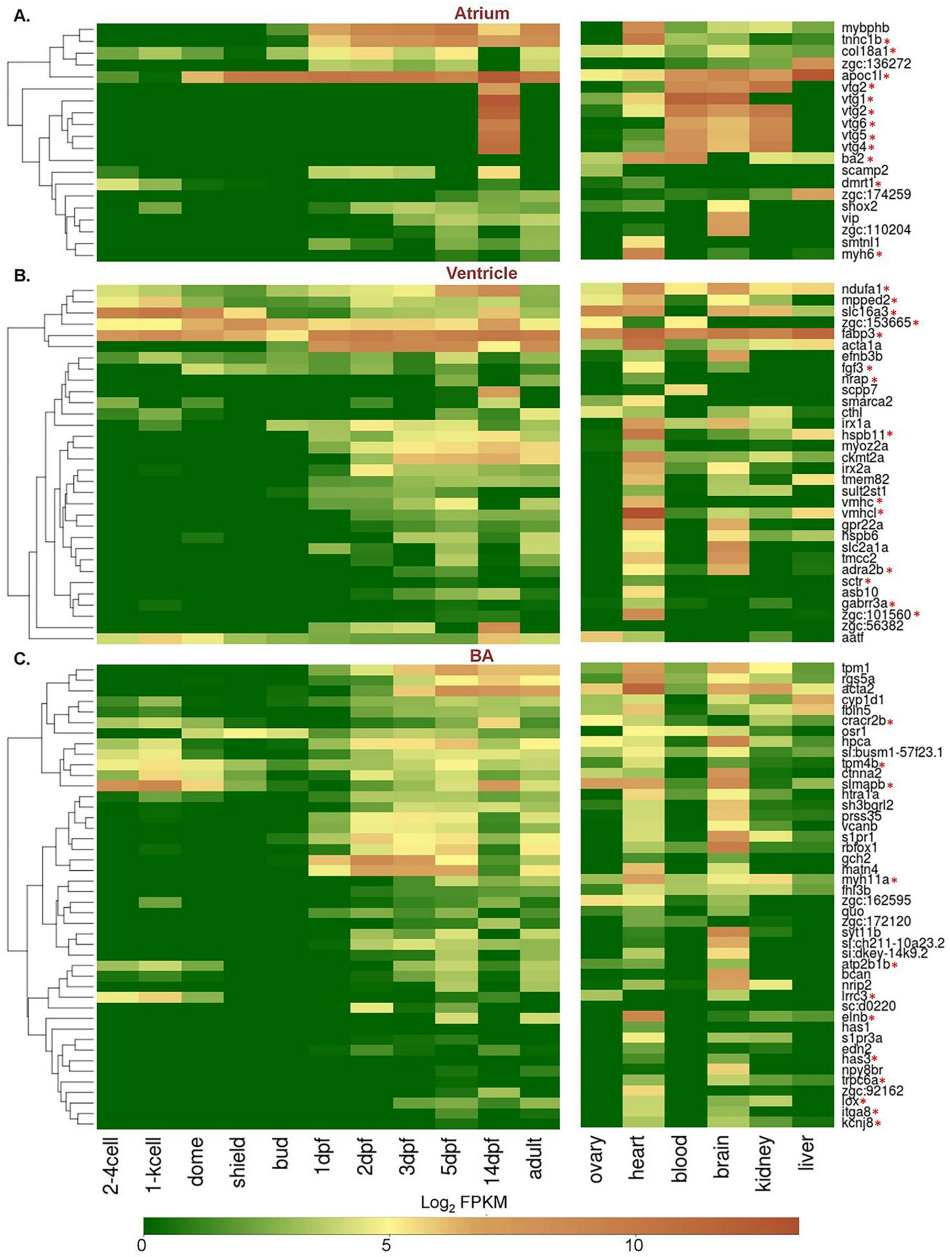
Developmental and adult tissue expression profiles of differentially expressed genes in the three cardiac chambers. The heat maps represent spatiotemporal expression profile of 96 chamber-restricted genes across 11 zebrafish developmental stages and 6 adult tissue types. The colour key represents transcripts in the range of 0 for genes with least expression to 10 for transcripts with maximum expression. Genes marked with asterisks (*) represent those mentioned in the text (Results & Discussion).

To understand the spatiotemporal expression pattern of the 96 cardiac chamber-restricted protein coding genes, log2-normalized FPKM values of these genes from 11 developmental stages (2–4 cell, 1000 cell, dome, shield, bud, 1dpf, 2dpf, 3dpf, 5 dpf, 14 dpf and adult) [45][46], zebrafish transcriptome sequencing project [PRJEB1986] and 6 tissue types (ovary, blood, brain, lungs, kidney, liver) were compared and are represented as a heat map (Fig 4).

In this analysis, we found that some genes display expression as early as 2 cell stage and thus may have a maternal contribution. Our analysis mapped 20 genes that were observed to be restricted to the atrium and two of these genes i.e, *doublesex and mab-3 related transcription factor 1* (*dmrt1*) *and collagen type XVIII*, *alpha 1* (*col18a1*) were found to express moderately at 2–4 cell stage and 1000 cell stages (mid-blastula transition), respectively. *DMRT1* in humans acts as a male-specific transcriptional regulator and has a key function in sex determination and differentiation [75,76]. There is no reported information on cardiac specific mutations linked with *COL18A1* in humans, however the gene is linked with Knobloch syndrome-1 [64].

Of the 32 genes that were differentially expressed in the ventricle, *mpped2*, *slc16a3*, *fabp3* and *zgc153665* showed an early development (2–4 cell stage) expression. Expression of the ventricle- restricted gene *nadh dehydrogenase* (*ubiquinone*) *1 alpha sub complex 1* (*ndufa 1*) was observed at 2–4 cell stage in early development, showed peaks between 5dpf and 14dpf and later was found in adult tissues such as ovary, brain, blood, kidney and liver. The human ortholog, *NDUFA1* binds several transcription factors, which are associated with cardiac as well as muscle-specific expressions [77]. Besides expressing in the ventricle, a few genes such as *solute carrier family 16* (*monocarboxylate transporter*), *member 3* (*slc16a3*) and *metallo-phospho-esterase domain containing 2b* (*mpped2*) (Fig 2) were also found to be expressed in the zebrafish brain, ovary and kidney (Fig 4). Similarly, the human ortholog, *MPEPED2*, has been reported to express in both heart and kidney [78].

Of the total 44 genes which showed restricted expression in the bulbus arteriosus, *cracr2b*, *tpm4b*, *atp2b1b* and *irrc3* showed a moderate expression as early by 2–4 cell stage. A high expression of *sarcolemma associated protein b* (*slmapb*) was observed at 2–4 cells to 1000 cell in addition to the bulbus arteriosus. Further analysis revealed a subset of genes which were silent during the early stages of development and instead displayed expression afterwards indicating function in later development (Fig 4). Such genes include *vtg1*, *vtg2*, *vtg4*, *ba2*, *vtg5* and *vtg6* in the atrium; *gabrr3a*, *adra2b*, *nrap*, *sctr*, and *zgc;101560* in the ventricle; *itga8*, *kcnj8*, *lox*, *trpc6a*, *has1*, *has3* and *elnb* in the bulbus arteriosus. Among the 96 differentially expressed genes analyzed, 9 genes (*acta1a*, *pkmt2a*, *vmhcl*, *vmhc*, *hspb1l*, *myhlla*, *zgc*:*101560*, *elnb* and *myh6*) were found to be highly restricted in heart tissue (Fig 4) and their expression was not detected in early developmental stages as discussed earlier. We also observed that the bulbus arteriosus expresses three ion channel genes including *potassium inwardly-rectifying channel*, *subfamily J*, *member 8* (*kcnj8*), *ATPase*, *Ca*^*2+*^ *transporting*, *plasma membrane 1b* (*atp2b1b*) and *transient receptor potential cation channel*, *subfamily C*, *member 6a* (*trpc6a*). Of these, *kcnj8* and *trpc6a* were detected only in the adult tissues (heart, brain and kidney) and not detected in early developmental stages, thus pointing to their role in adult organ function.

### Discovery of novel protein-coding gene loci

In addition to studying expression profiles of known protein coding genes in the three cardiac chambers, we also attempted to identify novel protein coding genes with predominant expression levels (FPKM > = 10) in the cardiac chambers. To identify novel genes, we used the aligned reads from each chamber to perform a *de novo* transcriptome assembly using Cufflinks. The chamber transcriptomes were then merged using Cuffmerge to generate a total of 13,197 transcripts (8,460 genes) with expression levels > = 10 FPKM in at least one chamber (Fig 1A). After removing the transcripts with at least 1bp overlap with all known gene loci included in the latest RefSeq and Ensembl (Zv9, v79) gene catalogues, we obtained a total of 1,645 transcripts (1,585 genes). We then excluded transcripts shorter than 200 bp and predicted ORFs for the remaining set. The transcripts with predicted ORF longer than 30 amino acids which could represent functional peptides were chosen for further analysis. To identify potential protein coding loci, 1,280 transcripts (1,223 genes) retained from the previous step were assessed for their coding potential using two tools, CPC and PhyloCSF using CPC score threshold > 1 and PhyloCSF score > 100. This step generated a final set of 34 novel putative protein coding transcripts (23 genes) all of which showed restricted expression in the ventricle (S3 Table).

### Ribosome profiling suggests protein coding potential of putative novel protein-coding genes

In an attempt to provide an independent assessment of coding capacity of the novel protein-coding (PC) gene catalogue identified in this study, we analyzed the data from a recently reported ribosome profiling study [48] that was performed across 8 stages of zebrafish development. We alloted a Translation Efficiency Score (TES) for all RefSeq genes (coding as well as non coding) along with the 34 novel predicted protein coding transcripts based on their estimated ribosomal occupancy and the results obtained are summarized in S4 Fig. The median TES profile for the novel protein coding genes (0.05) was found to be comparable, if not higher than that of protein coding RefSeq genes (0.04), and significantly higher than that of non coding RefSeq genes (0.005).

### Developmental expression profiles of cardiac chamber-restricted putative novel protein coding genes

**Fig 5:**
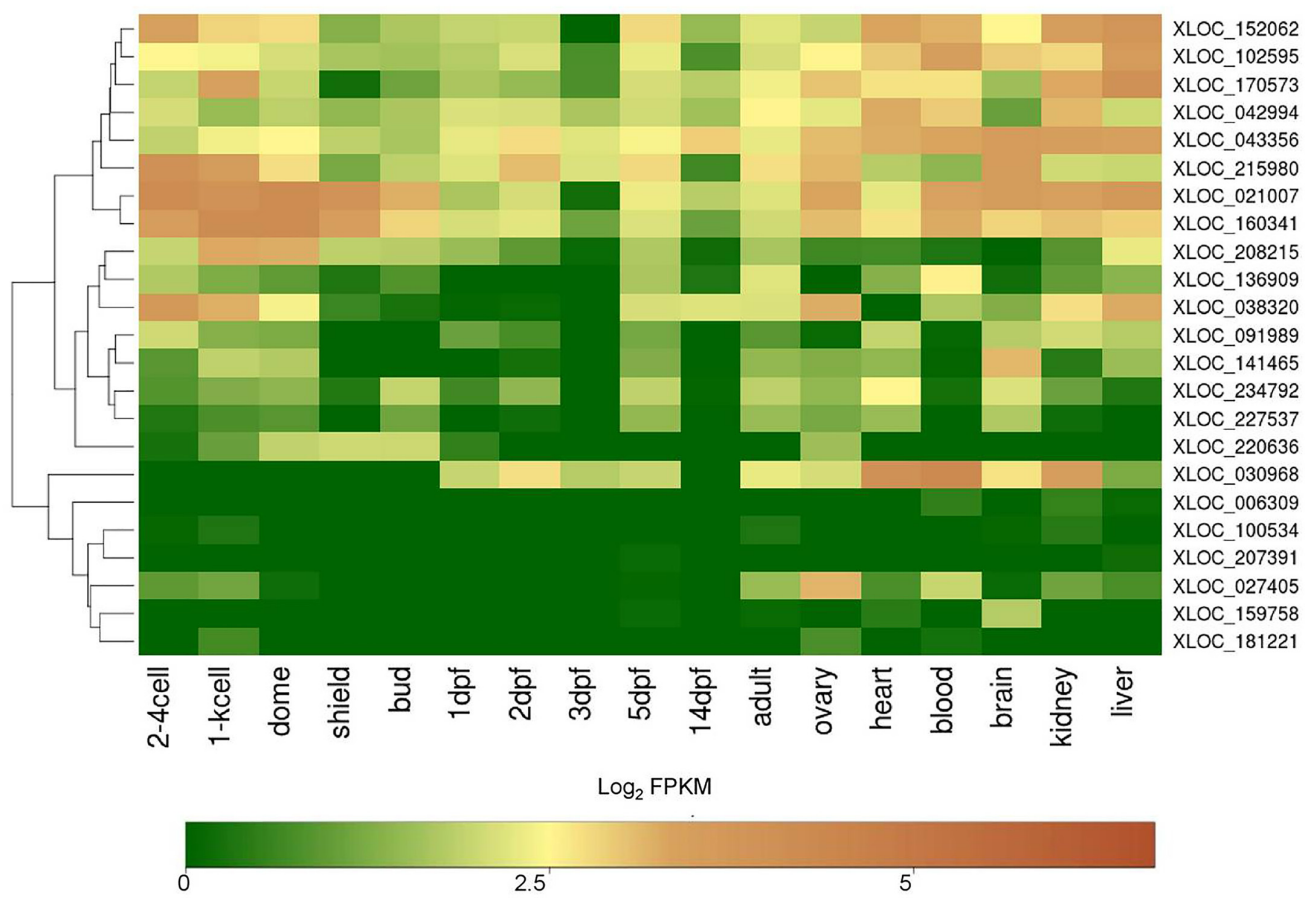
Developmental and adult tissue expression profile of the putative novel protein coding genes identified in the ventricular chamber. The heat maps represent spatiotemporal expression profile of 23 ventricle-restricted putative novel protein coding genes across 11 zebrafish developmental stages and 6 adult tissue types. The gene expression data for the embryonic and larval developmental stages were obtained from previous published studies (Ulitsky et al, 2011; Pauli et al, 2012) that utilized whole embryos or larvae. In contrast our dataset was obtained exclusively from the adult heart tissue. Thus, the expression profiles of the novel ventricular-restricted genes may not reflect in parallel to the early ventricular developmental hallmarks. The colour key represents transcripts in the range of 0 for genes with least expression to 5 for transcripts with maximum expression.

To understand spatiotemporal expression pattern of the 23 cardiac chamber-restricted putative novel protein coding genes, we plotted a heat map representing the log2-normalized FPKM values of the genes from 11 developmental stages and 6 tissue types as discussed earlier (Fig 5). We observed a spatiotemporal expression pattern for the putative novel protein coding genes across various developmental stages. Of the 23 genes analyzed, 5 genes (XLOC_152062, XLOC_ 215980, XLOC_021007, XLOC_160341, and XLOC_038320) displayed higher expression in the early developmental time points whereas 9 genes (XLOC_152062, XLOC_102595, XLOC_042994, XLOC_030968, XLOC_170573, XLOC_043356, XLOC_215980, XLOC_160341 and XLOC_021007) exhibited higher expression in the adult tissues (Fig 5).

### Validation of differentially expressed putative novel protein coding genes in cardiac chambers using qRT-PCR and whole mount *in situ* hybridization (WISH)

**Fig 6:**
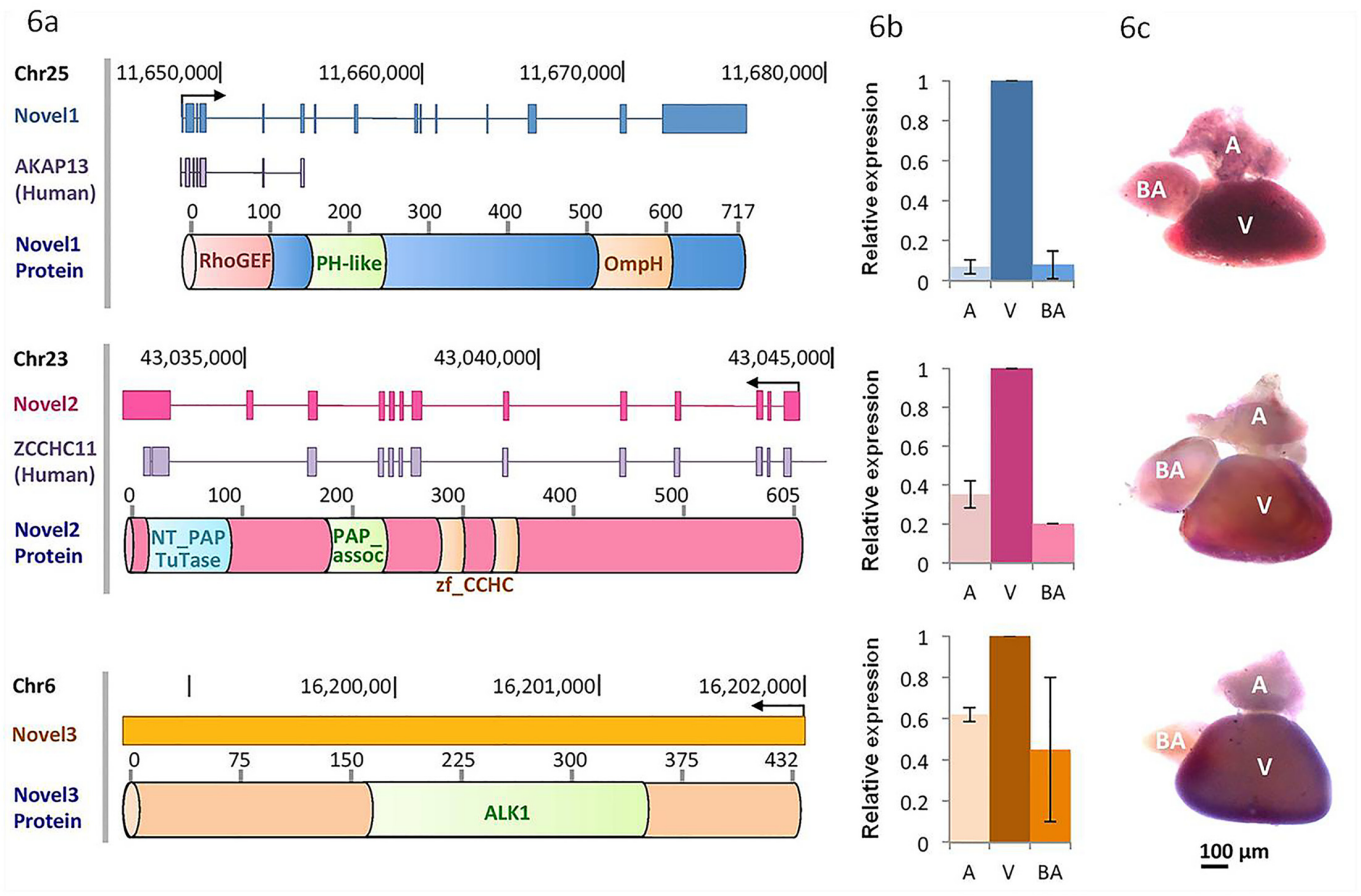
Experimental validation of top ventricle-restricted putative novel protein coding genes. (A) Putative exon structure, human protein homologs and protein domain architecture of the candidate novel protein coding genes. (B) Validation of ventricle-restricted protein coding gene candidates using qRT-PCR. (C) Validation of ventricle-restricted protein coding gene candidates using WISH. XLOC_170573 (Novel 1), XLOC_159758 (Novel 2) and XLOC_215980 (Novel 3) show restricted expression in the ventricle. Scale bar: 100μm. A (Atrium); V (Ventricle) and BA (Bulbous arteriosus).

To further validate the putative novel protein coding genes identified in our analysis, we selected three novel genes (XLOC_170573, XLOC_215980 and XLOC_159758) from the list of 23 putative protein coding genes (S3 Table) and performed qRT PCR and *in situ* hybridization. One of the genes, XLOC_170573 (Novel 1), which lies on chromosome 25, shows homology with the human protein *AKAP13* (A Kinase (*PRKA*) Anchor Protein 13), a RhoA GTPase-specific Guanine Exchange Factor (Fig 6A). *AKAP13* gene has been shown to have a role in cardiac development in mice [79]. XLOC_159758 (Novel 2) gene resides on chromosome 23 and was found to be homologous to the human protein, ZCCHC11 (Zinc Finger, CCHC Domain Containing 11). The third candidate gene, XLOC_215980 (Novel 3), resides on chromosome 6 and contains ALK (Anaplastic Lymphoma Kinase) domain, a tyrosine kinase receptor which has profound implications in multiple cancer types in humans [80] [81]. QRT-PCR analysis revealed comparatively restricted expression of all the three candidate genes in the ventricle. *In situ* hybridization further verified the restricted expression of these putative novel protein coding genes in the ventricle. Thus, we were able to verify the ventricle-restricted expression for these novel protein coding genes. (Fig 6B and 6C).

## Discussion

In this study, we have performed a comprehensive transcriptional profiling of the three cardiac chambers in zebrafish and identified genes that are involved in development and specification of adult heart chambers. Of the total volume (710 million reads) of data generated, approximately 84% of the sequencing reads mapped back to the reference genome suggesting that the data was of higher depth with a greater probability of most rare or less abundant transcripts being captured. Our study catalogued 13,396 protein coding genes, which were found to express in the cardiac chambers. Of these 7,260 proteins coding genes displayed significantly higher expression in the three chambers.

Diversity in gene expression between the chambers (atrium, ventricle and bulbus arteriosus) was well represented. The number of genes expressed in the atrium (5,951) and the bulbus arteriosus (5,823) was comparable whereas, the ventricle expressed a slightly larger repertoire (6,359). We notice that a subset of genes show overlap between two of the three chambers with 300 genes commonly expressed in both atrium and bulbous arteriosus, 529 genes shared between atrium and ventricle and 257 genes collectively expressing in ventricle and bulbus arteriosus (Fig 1B). We speculate that this subset of genes is primarily those that have similar or complementary functions across more than one chamber.

This study revealed 96 known protein coding genes that displayed chamber-restricted gene expression (20 in atrium, 32 in ventricle and 44 in bulbus arteriosus). The zebrafish atrium, in addition to genes involved in muscle contraction and calcium ion transport, also displayed representation of genes involved in lipid transport. The ventricle was observed to be enriched in a broader spectrum of genes, those representing transport proteins, receptor molecules, kinases and heat shock proteins apart from contractile proteins. The transcriptome signature in the bulbous arteriosus was dominated by contractile genes and calcium binding proteins. The specific enrichment of these protein coding genes in a particular chamber undercsores the fact that differences in gene expression govern the key functions among different cardiac chambers.

The developmental expression profiles of the 96 cardiac chamber genes across 11 developmental stages and 6 tissue types was analyzed and parallels in expression patterns between humans and zebrafish could be observed. One example is that of the zebrafish *mpped2* gene which shows expression in kidney apart from the heart. The human ortholog, *MPPED2* is also reported to show a kidney specific expression [78].

We demonstrate the approach of *de novo* transcript assembly to identify 23 putative novel protein coding genes, the expression of which was found to be restricted to the ventricle only. CPC and PhyloCSF values as well as ribosome profiling data suggest a strong coding potential for these 23 genes. We further verified the ventricle-restricted expression for a subset of these novel genes using *in situ* hybridization and qRT-PCR. The developmental expression analysis of these novel genes reflects that a few genes express in a broader time window during early development (2–4 cell stage to bud stage) and in adult stages show expression across multiple organs /tissues. We observed that for a majority of these 23 genes, expression levels were higher in adult organs as compared to early developmental stages.

A total of 68 genes that are observed to be differentially expressed in the zebrafish cardiac chambers, have a human ortholog reported (Fig 2; S2 Table). Of these, 25 genes are reported to be associated with various human diseases including 5 genes (*MYH6*, *MYH7*, *MYOZ2*, *TPM1* and *TNNC1*) to be linked with cardiac related disorders. The human *MYH6* gene is expressed in atrial tissue and when mutated, results in dilated cardiomyopathy 1EE [82], familial hypertrophic cardiomyopathy 14 [83] and atrial septal defects 3 [84]. Similarly, mutations in the classic ventricle marker, *MYH7* (human ortholog of zebrafish *vmhc* gene) are reported to result in familial dilated cardiomyopathy 1S and hypertrophic cardiomyopathy 1 [85]. Recently, a study on transcriptome analysis of the zebrafish heart has demonstrated that majority of the human dilated cardiomyopathy linked genes displayed a zebrafish ortholog [86]. In our dataset, *MYOZ2* (*myoz2a*) [87], *TPM1* (*tpm1*) [88] and *TNNC1* (*tnnc1a*) [62] have been identified which are previously reported to be linked with diverse forms of dilated and hypertrophic cardiomyopathy. Thus, the current cardiac transcriptome expression dataset from zebrafish may serve as a template for understanding genes involved in human cardiac development and disease.

We would like to highlight that some of the genes that are important in cardiac function have not been included in our transcriptome catalogue. For example, in agreement with a previous study by Tabibiazar and co-workers [89], our analysis generated differentially expressed subset of protein coding genes such as *LIM domain binding 3b* (*ldb3*), *nk2 homeobox 5* (*nkx2*.*5*), *wingless-type MMTV integration site family*, *member 2bb* (*wnt2bb*) and *t-box 5b* (*tbx5b*). However, these genes were omitted from our transcriptome catalogue as the fold change and expression level of these genes across the chambers could not qualify the cut off standards set for analysis in our study.

Another example of omission is that of *SERCA* or *atp2a2*, that was observed to express 3 fold in the ventricle when compared to the atrium and 60 fold when compared to the bulbus arteriosus. SERCAs are closely related to the plasma membrane Ca^2+^-ATPases and function as intracellular pumps localized in the sarcoplasmic reticulum of muscle cells. Human ortholog, *ATP2A2* (OMIM: 108740) is reported to cause an autosomal dominant disorder called, Darier disease [90] and null mutations in mouse demonstrate reduced myocyte contractility [90,91]. Another gene that is not a part of this transcriptome is *pyruvate kinase*, *muscle b* (*pkmb*).

Based on the gene ontology analysis, we observed that the ventricle expresses genes majorly involved in functions including, contraction, calcium signalling pathways and energy production, whereas, the atrium majorly expresses genes involved in metabolism and ligand receptors signalling (S4 Table). On the other hand, we found that the bulbus arteriosus expresses genes which are majorly linked to adhesion and contraction (S4 Table). We additionally identified 23 putative novel protein coding genes, which have not been annotated before in zebrafish and have opened the possibility of exploring the role of these genes in ventricular function.

This study provides a comprehensive catalogue of putative cardiac genes that express in the three chambers of the zebrafish heart. We describe the methods and applications of transcriptome analysis in zebrafish cardiac chambers using RNA-sequencing. Our work highlights the power of RNA-Sequencing in *de novo* transcriptome profiling [92]. Our study revealed differential expression of genes that may be linked to vital aspects of establishing the structural organization and functional resolution within the three zebrafish cardiac chambers. We hope this comprehensive catalogue of cardiac chamber-restricted gene expression will add to the ever growing information on the zebrafish transcriptome and will serve as a basal atlas for further investigations into the mechanisms that regulate cardiac function and chamber morphogenesis in zebrafish.

## Supporting Information

S1 Fig The zebrafish heart anatomy. (a) Cartoon of the adult zebrafish heart, (b) Dissected adult heart and the three chambers used for the present transcriptome analysis, (c) Atrium (d) Ventricle, (e) Bulbus arteriosus. Scale bar: 500μm.
(TIF)

S2 Fig RNA isolated from the three cardiac chambers. (1) Atrium, (2) Bulbus arteriosus and (3) Ventricle used for the present transcriptome analysis. The RNA was isolated from pooled samples of individual chambers dissected from 15 animals.
(TIF)

S3 Fig Real time qRT-PCR assay for cardiac chamber-restricted marker gene expression. The dissected cardiac chambers show specific marker genes expression. (A) Atrium, (V) Ventricle and (BA) Bulbus arteriosus. (a) *myh6* and (b) *vmhc* were used as marker genes for the atrium and the ventricle respectively. The markers show restricted expression in individual specific chambers and are either not expressed or minimally expressed in other chambers.
(TIF)

S4 Fig Comparison of translational efficiency scores of the putative novel protein coding transcripts with respect to RefSeq protein coding and non coding transcripts. The box plot depicts the distribution of Translation Efficiency Scores (TES) across putative novel protein coding transcripts (34) identified in this study and RefSeq genes (coding and non coding). Centre lines show the median; box limits indicate the 25th and 75th percentiles; whiskers extend till the 5th and 95th percentiles.
(TIF)

S5 Fig Experimental validation of chamber-restricted differentially expressing protein coding genes. Ventricle-restricted genes (a) *irx1a* and (b) *ldb3a*, Atrium- restricted gene (c) *vtg2*, Bulbus arteriosus-restricted gene (d) *rgs5a*. (A) Atrium, (V) Ventricle and (BA) Bulbus arteriosus. Scale bar: 100 μm. See text for detailed information.
(TIF)

S1 Table List of gene primers. (DOCX)

S2 Table List of genes differentially expressed in the zebrafish cardiac chambers and details on zebrafish mutants and human disease gene orthologs. (DOCX)

S3 Table List of all putative novel protein coding gene loci identified in the present study. (DOCX)

S4 Table KEGG pathway analysis. (DOCX)

## References

[1] Bakkers J. Zebrafish as a model to study cardiac development and human cardiac disease. Cardiovasc Res. 2011; 91: 279–288. 10.1093/cvr/cvr098 21602174 PMC3125074

[2] Nemtsas P, Wettwer E, Christ T, Weidinger G, Ravens U. Adult zebrafish heart as a model for human heart? An electrophysiological study. J Mol Cell Cardiol. 2010; 48: 161–171. 10.1016/j.yjmcc.2009.08.034 19747484

[3] Laflamme MA, Murry CE. Heart regeneration. Nature. 2011; 19;473: 326–335. 10.1038/nature10147 21593865 PMC4091722

[4] Liu Y, Morley M, Brandimarto J, Hannenhalli S, Hu Y, Ashley EA, et al RNA-Seq identifies novel myocardial gene expression signatures of heart failure. Genomics. 2015; 105: 83–89. 10.1016/j.ygeno.2014.12.002 25528681 PMC4684258

[5] Gong W, Koyano-Nakagawa N, Li T, Garry DJ. Inferring dynamic gene regulatory networks in cardiac differentiation through the integration of multi-dimensional data. BMC Bioinformatics. 2015; 16:74 10.1186/s12859-015-0460-0: 74–0460. 25887857 PMC4359553

[6] Pfaffl MW. A new mathematical model for relative quantification in real-time RT-PCR. Nucleic Acids Res. 2001; 29: e45 11328886 10.1093/nar/29.9.e45 PMC55695

[7] Iruretagoyena JI, Davis W, Bird C, Olsen J, Radue R, Teo BA, et al Metabolic gene profile in early human fetal heart development. Mol Hum Reprod. 2014; 20: 690–700. 10.1093/molehr/gau026 24674993 PMC11514182

[8] Rodius S, Nazarov PV, Nepomuceno-Chamorro IA, Jeanty C, Gonzalez-Rosa JM, Ibberson M, et al Transcriptional response to cardiac injury in the zebrafish: systematic identification of genes with highly concordant activity across in vivo models. BMC Genomics. 2014; 15:852 10.1186/1471-2164-15-852: 852–15. 25280539 PMC4197235

[9] Lescroart F, Chabab S, Lin X, Rulands S, Paulissen C, Rodolosse A, et al Early lineage restriction in temporally distinct populations of Mesp1 progenitors during mammalian heart development. Nat Cell Biol. 2014; 16: 829–840. 10.1038/ncb3024 25150979 PMC6984965

[10] Zhou J, Gao J, Liu Y, Gu S, Zhang X, An X, et al Human atrium transcript analysis of permanent atrial fibrillation. Int Heart J. 2014; 55: 71–77. 24463922 10.1536/ihj.13-196

[11] Chen J, Wang HY, Zeng CY. Transcriptome network analysis of potential candidate genes for heart failure. Genet Mol Res. 2013; 12: 4687–4697. 10.4238/2013.October.18.7 24222245

[12] Dang MQ, Zhao XC, Lai S, Wang X, Wang L, Zhang YL, et al Gene expression profile in the early stage of angiotensin II-induced cardiac remodeling: a time series microarray study in a mouse model. Cell Physiol Biochem. 2015; 35: 467–476. 10.1159/000369712 25613478

[13] Geng J, Picker J, Zheng Z, Zhang X, Wang J, Hisama F, et al Chromosome microarray testing for patients with congenital heart defects reveals novel disease causing loci and high diagnostic yield. BMC Genomics. 2014; 15:1127 10.1186/1471-2164-15-1127: 1127–15. 25516202 PMC4378009

[14] Liu Y, Li G, Lu H, Li W, Li X, Liu H, et al Expression profiling and ontology analysis of long noncoding RNAs in post-ischemic heart and their implied roles in ischemia/reperfusion injury. Gene. 2014; 543: 15–21. 10.1016/j.gene.2014.04.016 24726549

[15] Liu H, Chen GX, Liang MY, Qin H, Rong J, Yao JP, et al Atrial fibrillation alters the microRNA expression profiles of the left atria of patients with mitral stenosis. BMC Cardiovasc Disord. 2014; 14:10 10.1186/1471-2261-14-10: 10–14. 24461008 PMC3909014

[16] Li D, Chen G, Yang J, Fan X, Gong Y, Xu G, et al Transcriptome analysis reveals distinct patterns of long noncoding RNAs in heart and plasma of mice with heart failure. PLoS One. 2013; 8: e77938 10.1371/journal.pone.0077938 24205036 PMC3812140

[17] Song G, Shen Y, Zhu J, Liu H, Liu M, Shen YQ, et al Integrated analysis of dysregulated lncRNA expression in fetal cardiac tissues with ventricular septal defect. PLoS One. 2013; 8: e77492 10.1371/journal.pone.0077492 24147006 PMC3797806

[18] Muthusamy S, DeMartino AM, Watson LJ, Brittian KR, Zafir A, Dassanayaka S, et al MicroRNA-539 is up-regulated in failing heart, and suppresses O-GlcNAcase expression. J Biol Chem. 2014; 289: 29665–29676. 10.1074/jbc.M114.578682 25183011 PMC4207981

[19] Franco D, Lamers WH, Moorman AF. Patterns of expression in the developing myocardium: towards a morphologically integrated transcriptional model. Cardiovasc Res. 1998; 38: 25–53. 9683906 10.1016/s0008-6363(97)00321-0

[20] Shendure J. The beginning of the end for microarrays? Nat Methods. 2008; 5: 585–587. 10.1038/nmeth0708-585 18587314

[21] Malone JH, Oliver B. Microarrays, deep sequencing and the true measure of the transcriptome. BMC Biol. 2011; 9:34 10.1186/1741-7007-9-34: 34–39. 21627854 PMC3104486

[22] Najmabadi H, Hu H, Garshasbi M, Zemojtel T, Abedini SS, Chen W, et al Deep sequencing reveals 50 novel genes for recessive cognitive disorders. Nature. 2011; 478: 57–63. 10.1038/nature10423 21937992

[23] Pan Q, Shai O, Lee LJ, Frey BJ, Blencowe BJ. Deep surveying of alternative splicing complexity in the human transcriptome by high-throughput sequencing. Nat Genet. 2008; 40: 1413–1415. 10.1038/ng.259 18978789

[24] Sultan M, Schulz MH, Richard H, Magen A, Klingenhoff A, Scherf M, et al A global view of gene activity and alternative splicing by deep sequencing of the human transcriptome. Science. 2008; 321: 956–960. 10.1126/science.1160342 18599741

[25] Yang KC, Yamada KA, Patel AY, Topkara VK, George I, Cheema FH, et al Deep RNA sequencing reveals dynamic regulation of myocardial noncoding RNAs in failing human heart and remodeling with mechanical circulatory support. Circulation. 2014; 129: 1009–1021. 10.1161/CIRCULATIONAHA.113.003863 24429688 PMC3967509

[26] Leptidis S, El AH, Lok SI, de WR, Olieslagers S, Kisters N, A deep sequencing approach to uncover the miRNOME in the human heart. PLoS One. 2013; 8: e57800 10.1371/journal.pone.0057800 23460909 PMC3583901

[27] Liu HL, Zhu JG, Liu YQ, Fan ZG, Zhu C, Qian LM. Identification of the microRNA expression profile in the regenerative neonatal mouse heart by deep sequencing. Cell Biochem Biophys. 2014; 70: 635–642. 10.1007/s12013-014-9967-7 24756729

[28] Bao ZZ, Bruneau BG, Seidman JG, Seidman CE, Cepko CL. Regulation of chamber-specific gene expression in the developing heart by Irx4. Science. 1999; 19;283: 1161–1164. 10024241 10.1126/science.283.5405.1161

[29] Yamagishi H, Yamagishi C, Nakagawa O, Harvey RP, Olson EN, Srivastava D. The combinatorial activities of Nkx2.5 and dHAND are essential for cardiac ventricle formation. Dev Biol. 2001; 239: 190–203. 11784028 10.1006/dbio.2001.0417

[30] Bruneau BG, Bao ZZ, Tanaka M, Schott JJ, Izumo S, Cepko CL. Cardiac expression of the ventricle-specific homeobox gene Irx4 is modulated by Nkx2-5 and dHand. Dev Biol. 2000; 217: 266–277. 10625552 10.1006/dbio.1999.9548

[31] Zhao XS, Gallardo TD, Lin L, Schageman JJ, Shohet RV. Transcriptional mapping and genomic analysis of the cardiac atria and ventricles. Physiol Genomics. 2002; 12: 53–60. 12502795 10.1152/physiolgenomics.00086.2002

[32] Sucov HM. Molecular insights into cardiac development. Annu Rev Physiol. 1998; 60:287–308.: 287–308. 9558465 10.1146/annurev.physiol.60.1.287

[33] Kaynak B, von HA, Mebus S, Seelow D, Hennig S, Vogel J, et al Genome-wide array analysis of normal and malformed human hearts. Circulation. 2003; 20;107: 2467–2474. 12742993 10.1161/01.CIR.0000066694.21510.E2

[34] Patowary A, Purkanti R, Singh M, Chauhan R, Singh AR, Swarnkar M, et al A sequence-based variation map of zebrafish. Zebrafish. 2013; 10: 15–20. 10.1089/zeb.2012.0848 23590399 PMC3629779

[35] Westerfield M. The zebrafish book. A guide for the laboratory use of zebrafish (*Danio rerio*). Eugene 2000 University of Oregon Press.

[36] Kaushik K, Leonard VE, Kv S, Lalwani MK, Jalali S, Patowary A, et al Dynamic expression of long non-coding RNAs (lncRNAs) in adult zebrafish. PLoS One. 2013; 8: e83616 10.1371/journal.pone.0083616 24391796 PMC3877055

[37] Bolger AM, Lohse M, Usadel B. Trimmomatic: a flexible trimmer for Illumina sequence data. Bioinformatics. 2014; 30: 2114–2120. 10.1093/bioinformatics/btu170 24695404 PMC4103590

[38] Dobin A, Davis CA, Schlesinger F, Drenkow J, Zaleski C, Jha S, Batut P, et al STAR: ultrafast universal RNA-seq aligner. Bioinformatics. 2013; 29: 15–21. 10.1093/bioinformatics/bts635 23104886 PMC3530905

[39] Trapnell C, Williams BA, Pertea G, Mortazavi A, Kwan G, van Baren MJ, et al Transcript assembly and quantification by RNA-Seq reveals unannotated transcripts and isoform switching during cell differentiation. Nat Biotechnol. 2010; 28: 511–515. 10.1038/nbt.1621 20436464 PMC3146043

[40] Rice P, Longden I, Bleasby A. EMBOSS: the European Molecular Biology Open Software Suite. Trends Genet. 2000; 16: 276–277. 10827456 10.1016/s0168-9525(00)02024-2

[41] Kong L, Zhang Y, Ye ZQ, Liu XQ, Zhao SQ, Wei L. CPC: assess the protein-coding potential of transcripts using sequence features and support vector machine. Nucleic Acids Res. 2007; 35: W345–W349. 17631615 10.1093/nar/gkm391 PMC1933232

[42] Lin MF, Jungreis I, Kellis M. PhyloCSF: a comparative genomics method to distinguish protein coding and non-coding regions. Bioinformatics. 2011; 27: i275–i282. 10.1093/bioinformatics/btr209 21685081 PMC3117341

[43] Eddy SR. Accelerated Profile HMM Searches. PLoS Comput Biol. 2011; 7: e1002195 10.1371/journal.pcbi.1002195 22039361 PMC3197634

[44] Punta M, Coggill PC, Eberhardt RY, Mistry J, Tate J, Boursnell C. The Pfam protein families database. Nucleic Acids Res. 2012; 40: D290–D301. 10.1093/nar/gkr1065 22127870 PMC3245129

[45] Pauli A, Valen E, Lin MF, Garber M, Vastenhouw NL, Levin JZ, et al Systematic identification of long noncoding RNAs expressed during zebrafish embryogenesis. Genome Res. 2012; 22: 577–591. 10.1101/gr.133009.111 22110045 PMC3290793

[46] Ulitsky I, Shkumatava A, Jan CH, Sive H, Bartel DP. Conserved function of lincRNAs in vertebrate embryonic development despite rapid sequence evolution. Cell. 2011; 147: 1537–1550. 10.1016/j.cell.2011.11.055 22196729 PMC3376356

[47] Wang J, Duncan D, Shi Z, Zhang B. WEB-based GEne SeT AnaLysis Toolkit (WebGestalt): update 2013. Nucleic Acids Res. 2013; 41: W77–W83. 10.1093/nar/gkt439 23703215 PMC3692109

[48] Chew GL, Pauli A, Rinn JL, Regev A, Schier AF, Valen E. Ribosome profiling reveals resemblance between long non-coding RNAs and 5' leaders of coding RNAs. Development. 2013; 140: 2828–2834. 10.1242/dev.098343 23698349 PMC3678345

[49] Ingolia NT, Lareau LF, Weissman JS. Ribosome profiling of mouse embryonic stem cells reveals the complexity and dynamics of mammalian proteomes. Cell. 2011; 147: 789–802. 10.1016/j.cell.2011.10.002 22056041 PMC3225288

[50] Quast C, Pruesse E, Yilmaz P, Gerken J, Schweer T, Yarza P, et al The SILVA ribosomal RNA gene database project: improved data processing and web-based tools. Nucleic Acids Res. 2013; 41: D590–D596. 10.1093/nar/gks1219 23193283 PMC3531112

[51] Langmead B, Salzberg SL. Fast gapped-read alignment with Bowtie 2. Nat Methods. 2012; 9: 357–359. 10.1038/nmeth.1923 22388286 PMC3322381

[52] Kim D, Pertea G, Trapnell C, Pimentel H, Kelley R, Salzberg SL. TopHat2: accurate alignment of transcriptomes in the presence of insertions, deletions and gene fusions. Genome Biol. 2013; 14: R36–14. 10.1186/gb-2013-14-4-r36 23618408 PMC4053844

[53] Cox MP, Peterson DA, Biggs PJ. SolexaQA: At-a-glance quality assessment of Illumina second-generation sequencing data. BMC Bioinformatics. 2010; 11:485 10.1186/1471-2105-11-485: 485–11. 20875133 PMC2956736

[54] Quinlan AR. BEDTools: The Swiss-Army Tool for Genome Feature Analysis. Curr Protoc Bioinformatics. 2014; 47:11.12.1–11.12.34. 10.1002/0471250953.bi1112s47: 11. PMC4213956 25199790

[55] Ruzicka L, Bradford YM, Frazer K, Howe DG, Paddock H, Ramachandran S, et al ZFIN, The zebrafish model organism database: Updates and new directions. Genesis. 2015; 20 10.1002/dvg.22868: 10. PMC4545674 26097180

[56] Hamosh A, Scott AF, Amberger JS, Bocchini CA, McKusick VA. Online Mendelian Inheritance in Man (OMIM), a knowledgebase of human genes and genetic disorders. Nucleic Acids Res. 2005; 33: D514–D517. 15608251 10.1093/nar/gki033 PMC539987

[57] Berdougo E, Coleman H, Lee DH, Stainier DY, Yelon D. Mutation of weak atrium/atrial myosin heavy chain disrupts atrial function and influences ventricular morphogenesis in zebrafish. Development. 2003; 130: 6121–6129. 14573521 10.1242/dev.00838

[58] Somi S, Houweling AC, Buffing AA, Moorman AF, Van Den Hoff MJ. Expression of cVg1 mRNA during chicken embryonic development. Anat Rec A Discov Mol Cell Evol Biol. 2003; 273: 603–608. 12808645 10.1002/ar.a.10070

[59] Thisse C, Thisse B. High-resolution in situ hybridization to whole-mount zebrafish embryos. Nat Protoc. 2008; 3: 59–69. 10.1038/nprot.2007.514 18193022

[60] Sogah VM, Serluca FC, Fishman MC, Yelon DL, Macrae CA, Mably JD. Distinct troponin C isoform requirements in cardiac and skeletal muscle. Dev Dyn. 2010; 239: 3115–3123. 10.1002/dvdy.22445 20925115 PMC2965274

[61] Hoffmann B, Schmidt-Traub H, Perrot A, Osterziel KJ, Gessner R. First mutation in cardiac troponin C, L29Q, in a patient with hypertrophic cardiomyopathy. Hum Mutat. 2001; 17: 524. 10.1002/humu.1143 11385718

[62] Mogensen J, Murphy RT, Kubo T, Bahl A, Moon JC, Klausen IC, et al Frequency and clinical expression of cardiac troponin I mutations in 748 consecutive families with hypertrophic cardiomyopathy. J Am Coll Cardiol. 2004; 44: 2315–2325. 15607392 10.1016/j.jacc.2004.05.088

[63] Wooldridge AA, Fortner CN, Lontay B, Akimoto T, Neppl RL, Facemire C. Deletion of the protein kinase A/protein kinase G target SMTNL1 promotes an exercise-adapted phenotype in vascular smooth muscle. J Biol Chem. 2008; 283: 11850–11859. 10.1074/jbc.M708628200 18310078 PMC2431077

[64] Sertie AL, Sossi V, Camargo AA, Zatz M, Brahe C, Passos-Bueno MR. Collagen XVIII, containing an endogenous inhibitor of angiogenesis and tumor growth, plays a critical role in the maintenance of retinal structure and in neural tube closure (Knobloch syndrome). Hum Mol Genet. 2000; 9: 2051–2058. 10942434 10.1093/hmg/9.13.2051

[65] Everett AW, Umeda PK, Sinha AM, Rabinowitz M, Zak R. Expression of myosin heavy chains during thyroid hormone-induced cardiac growth. Fed Proc. 1986; 45: 2568–2572. 3758376

[66] Posch MG, Waldmuller S, Muller M, Scheffold T, Fournier D, Andrade-Navarro MA, et al Cardiac alpha-myosin (MYH6) is the predominant sarcomeric disease gene for familial atrial septal defects. PLoS One. 2011; 6: e28872 10.1371/journal.pone.0028872 22194935 PMC3237499

[67] Holm H, Gudbjartsson DF, Sulem P, Masson G, Helgadottir HT, Zanon C, et al A rare variant in MYH6 is associated with high risk of sick sinus syndrome. Nat Genet. 2011; 43: 316–320. 10.1038/ng.781 21378987 PMC3066272

[68] Laing NG, Dye DE, Wallgren-Pettersson C, Richard G, Monnier N, Lillis S, et al Mutations and polymorphisms of the skeletal muscle alpha-actin gene (ACTA1). Hum Mutat. 2009; 30: 1267–1277. 10.1002/humu.21059 19562689 PMC2784950

[69] Klein SC, Haas RC, Perryman MB, Billadello JJ, Strauss AW. Regulatory element analysis and structural characterization of the human sarcomeric mitochondrial creatine kinase gene. J Biol Chem. 1991; 266: 18058–18065. 1917943

[70] Pipkin W, Johnson JA, Creazzo TL, Burch J, Komalavilas P, Brophy C. Localization, macromolecular associations, and function of the small heat shock-related protein HSP20 in rat heart. Circulation. 2003; 107: 469–476. 12551873 10.1161/01.cir.0000044386.27444.5a

[71] Tessier DJ, Komalavilas P, Panitch A, Joshi L, Brophy CM. The small heat shock protein (HSP) 20 is dynamically associated with the actin cross-linking protein actinin. J Surg Res. 2003; 111: 152–157. 12842460 10.1016/s0022-4804(03)00113-6

[72] Zhang R, Xu X. Transient and transgenic analysis of the zebrafish ventricular myosin heavy chain (vmhc) promoter: an inhibitory mechanism of ventricle-specific gene expression. Dev Dyn. 2009; 238: 1564–1573. 10.1002/dvdy.21929 19322764 PMC2756512

[73] Seidman JG, Seidman C. The genetic basis for cardiomyopathy: from mutation identification to mechanistic paradigms. Cell. 2001; 104: 557–567. 11239412 10.1016/s0092-8674(01)00242-2

[74] Thisse B, Heyer V, Lux A, Alunni V, Degrave A, Seiliez I, et al Spatial and temporal expression of the zebrafish genome by large-scale in situ hybridization screening. Methods Cell Biol. 2004; 77:505–19.: 505–519. 15602929 10.1016/s0091-679x(04)77027-2

[75] Raymond CS, Shamu CE, Shen MM, Seifert KJ, Hirsch B, Hodgkin J, et al Evidence for evolutionary conservation of sex-determining genes. Nature. 1998; 391: 691–695. 9490411 10.1038/35618

[76] Ying M, Chen B, Tian Y, Hou Y, Li Q, Shang X, et al Nuclear import of human sexual regulator DMRT1 is mediated by importin-beta. Biochim Biophys Acta. 2007; 1773: 804–813. 17459496 10.1016/j.bbamcr.2007.03.006

[77] Zhuchenko O, Wehnert M, Bailey J, Sun ZS, Lee CC. Isolation, mapping, and genomic structure of an X-linked gene for a subunit of human mitochondrial complex I. Genomics. 1996; 37: 281–288. 8938439 10.1006/geno.1996.0561

[78] Pattaro C, Kottgen A, Teumer A, Garnaas M, Boger CA, Fuchsberger C, et al Genome-wide association and functional follow-up reveals new loci for kidney function. PLoS Genet. 1996; 2012; 8: e1002584 10.1371/journal.pgen.1002584 22479191 PMC3315455

[79] Mayers CM, Wadell J, McLean K, Venere M, Malik M, Shibata T, et al The Rho guanine nucleotide exchange factor AKAP13 (BRX) is essential for cardiac development in mice. J Biol Chem. 2010; 285: 12344–12354. 10.1074/jbc.M110.106856 20139090 PMC2852973

[80] Kwak EL, Bang YJ, Camidge DR, Shaw AT, Solomon B, Maki RG, et al Anaplastic lymphoma kinase inhibition in non-small-cell lung cancer. N Engl J Med. 2010; 363: 1693–1703. 10.1056/NEJMoa1006448 20979469 PMC3014291

[81] Koivunen JP, Mermel C, Zejnullahu K, Murphy C, Lifshits E, Holmes AJ, et al EML4-ALK fusion gene and efficacy of an ALK kinase inhibitor in lung cancer. Clin Cancer Res. 2008; 14: 4275–4283. 10.1158/1078-0432.CCR-08-0168 18594010 PMC3025451

[82] Carniel E, Taylor MR, Sinagra G, Di LA, Ku L, Fain PR, et al Alpha-myosin heavy chain: a sarcomeric gene associated with dilated and hypertrophic phenotypes of cardiomyopathy. Circulation. 2005; 112: 54–59. 15998695 10.1161/CIRCULATIONAHA.104.507699

[83] Niimura H, Patton KK, McKenna WJ, Soults J, Maron BJ, Seidman JG, et al Sarcomere protein gene mutations in hypertrophic cardiomyopathy of the elderly. Circulation. 2002; 105: 446–451. 11815426 10.1161/hc0402.102990

[84] Ching YH, Ghosh TK, Cross SJ, Packham EA, Honeyman L, Loughna S, et al Mutation in myosin heavy chain 6 causes atrial septal defect. Nat Genet. 2005; 37: 423–428. 15735645 10.1038/ng1526

[85] Kamisago M, Sharma SD, DePalma SR, Solomon S, Sharma P, McDonough B, et al Mutations in sarcomere protein genes as a cause of dilated cardiomyopathy. N Engl J Med. 2000; 343: 1688–1696. 11106718 10.1056/NEJM200012073432304

[86] Shih YH, Zhang Y, Ding Y, Ross CA, Li H, Olson TM, et al Cardiac transcriptome and dilated cardiomyopathy genes in zebrafish. Circ Cardiovasc Genet. 2015; 8: 261–269. 10.1161/CIRCGENETICS.114.000702 25583992 PMC4406804

[87] Osio A, Tan L, Chen SN, Lombardi R, Nagueh SF, Shete S, et al Myozenin 2 is a novel gene for human hypertrophic cardiomyopathy. Circ Res. 2007; 100: 766–768. 17347475 10.1161/01.RES.0000263008.66799.aa PMC2775141

[88] Watkins H, McKenna WJ, Thierfelder L, Suk HJ, Anan R, O'Donoghue A, et al Mutations in the genes for cardiac troponin T and alpha-tropomyosin in hypertrophic cardiomyopathy. N Engl J Med. 1995; 20;332: 1058–1064. 7898523 10.1056/NEJM199504203321603

[89] Tabibiazar R, Wagner RA, Liao A, Quertermous T. Transcriptional profiling of the heart reveals chamber-specific gene expression patterns. Circ Res. 2003; 93: 1193–1201. 14576202 10.1161/01.RES.0000103171.42654.DD

[90] Sakuntabhai A, Ruiz-Perez V, Carter S, Jacobsen N, Burge S, Monk S, et al Mutations in ATP2A2, encoding a Ca2+ pump, cause Darier disease. Nat Genet. 1999; 21: 271–277. 10080178 10.1038/6784

[91] Ji Y, Lalli MJ, Babu GJ, Xu Y, Kirkpatrick DL, Liu LH, et al Disruption of a single copy of the SERCA2 gene results in altered Ca2+ homeostasis and cardiomyocyte function. J Biol Chem. 2000; 275: 38073–38080. 10970890 10.1074/jbc.M004804200

[92] Wang Z, Gerstein M, Snyder M. RNA-Seq: a revolutionary tool for transcriptomics. Nat Rev Genet. 2009; 10: 57–63. 10.1038/nrg2484 19015660 PMC2949280

