# Supplementary figures and images for: Chamber Specific Gene Expression Landscape of the Zebrafish Heart
Source: PLoS One. 2016 Jan 27;11(1):e0147823. doi: 10.1371/journal.pone.0147823 (PMC4729522; doi:10.1371/journal.pone.0147823)

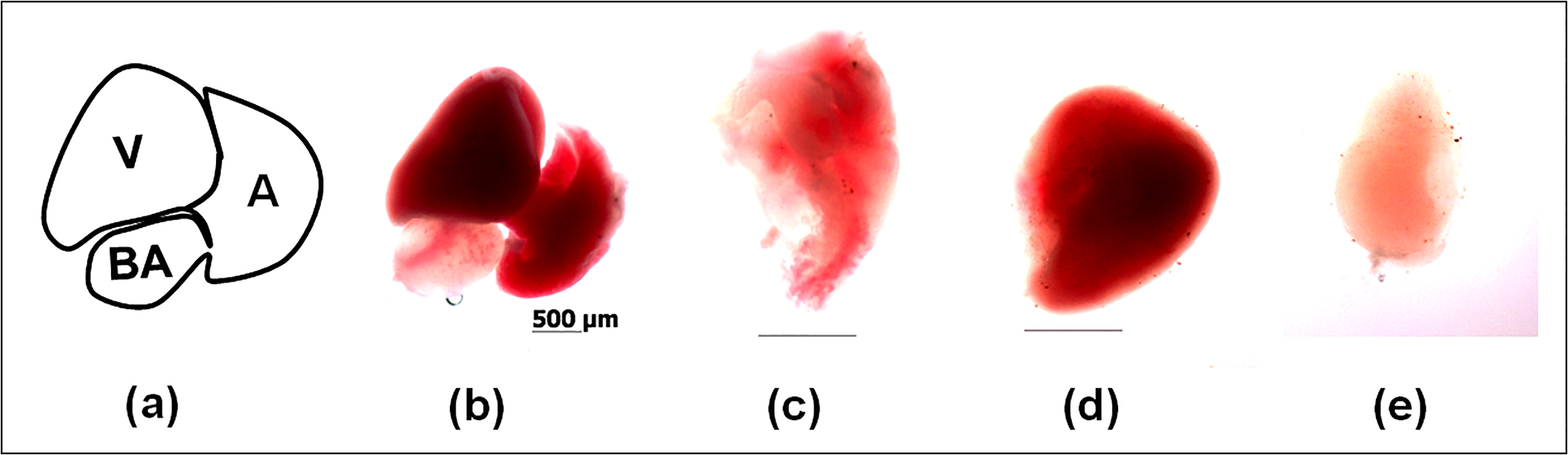

Supplement: S1 Fig — (a) Cartoon of the adult zebrafish heart, (b) Dissected adult heart and the three chambers used for the present transcriptome analysis, (c) Atrium (d) Ventricle, (e) Bulbus arteriosus. Scale bar: 500μm. (TIF) [file pone.0147823.s001.tif]

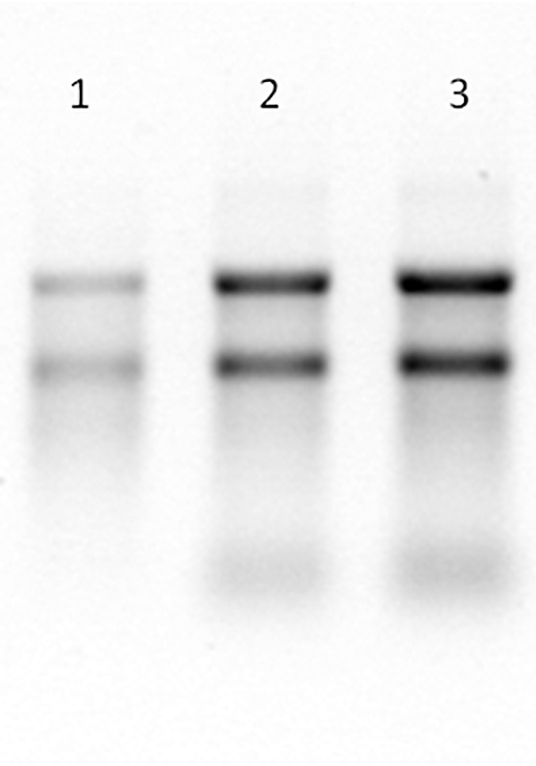

Supplement: S2 Fig — (1) Atrium, (2) Bulbus arteriosus and (3) Ventricle used for the present transcriptome analysis. The RNA was isolated from pooled samples of individual chambers dissected from 15 animals. (TIF) [file pone.0147823.s002.tif]

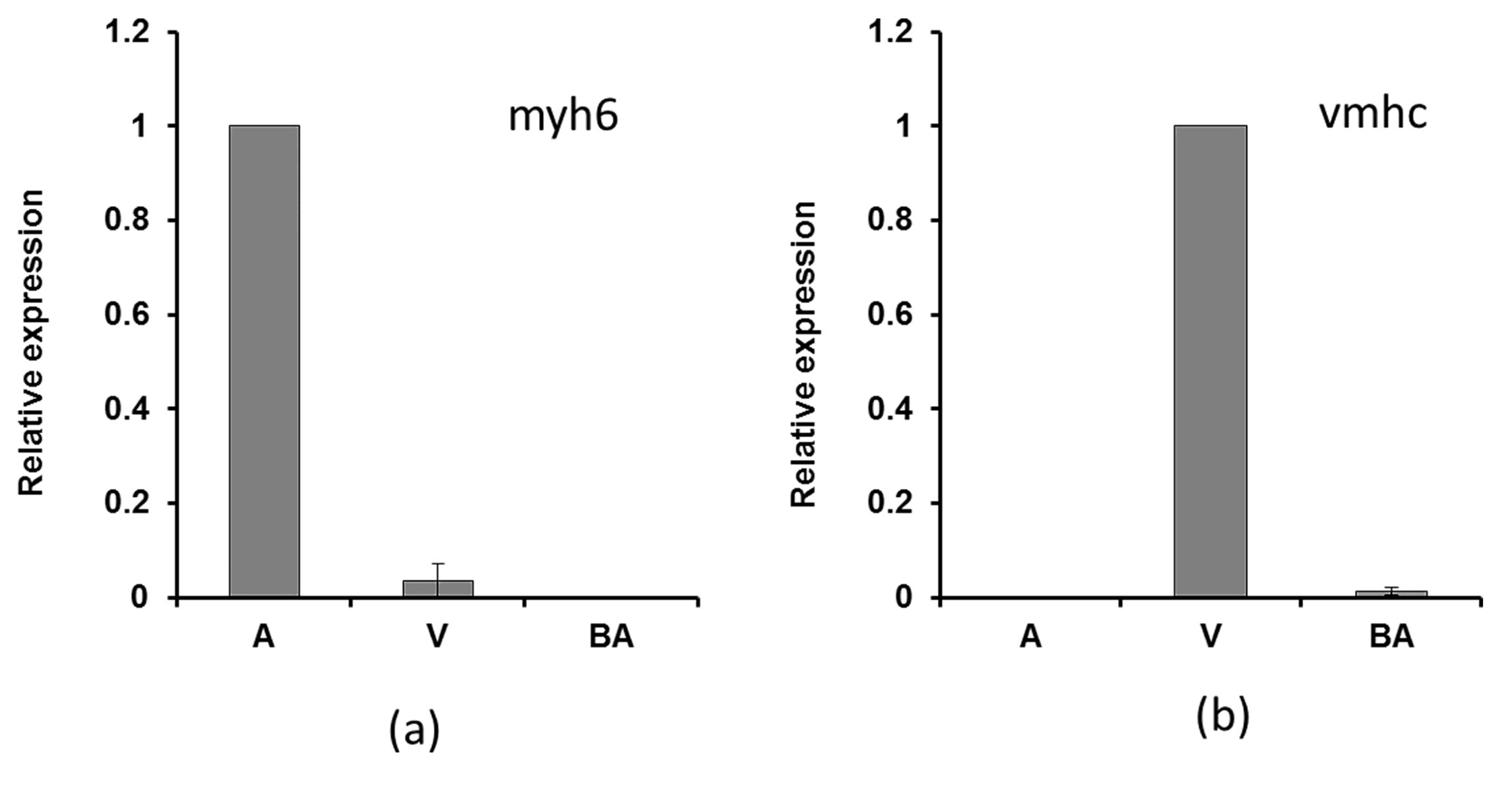

Supplement: S3 Fig — The dissected cardiac chambers show specific marker genes expression. (A) Atrium, (V) Ventricle and (BA) Bulbus arteriosus. (a) myh6 and (b) vmhc were used as marker genes for the atrium and the ventricle respectively. The markers show restricted expression in individual specific chambers and are either not expressed or minimally expressed in other chambers. (TIF) [file pone.0147823.s003.tif]

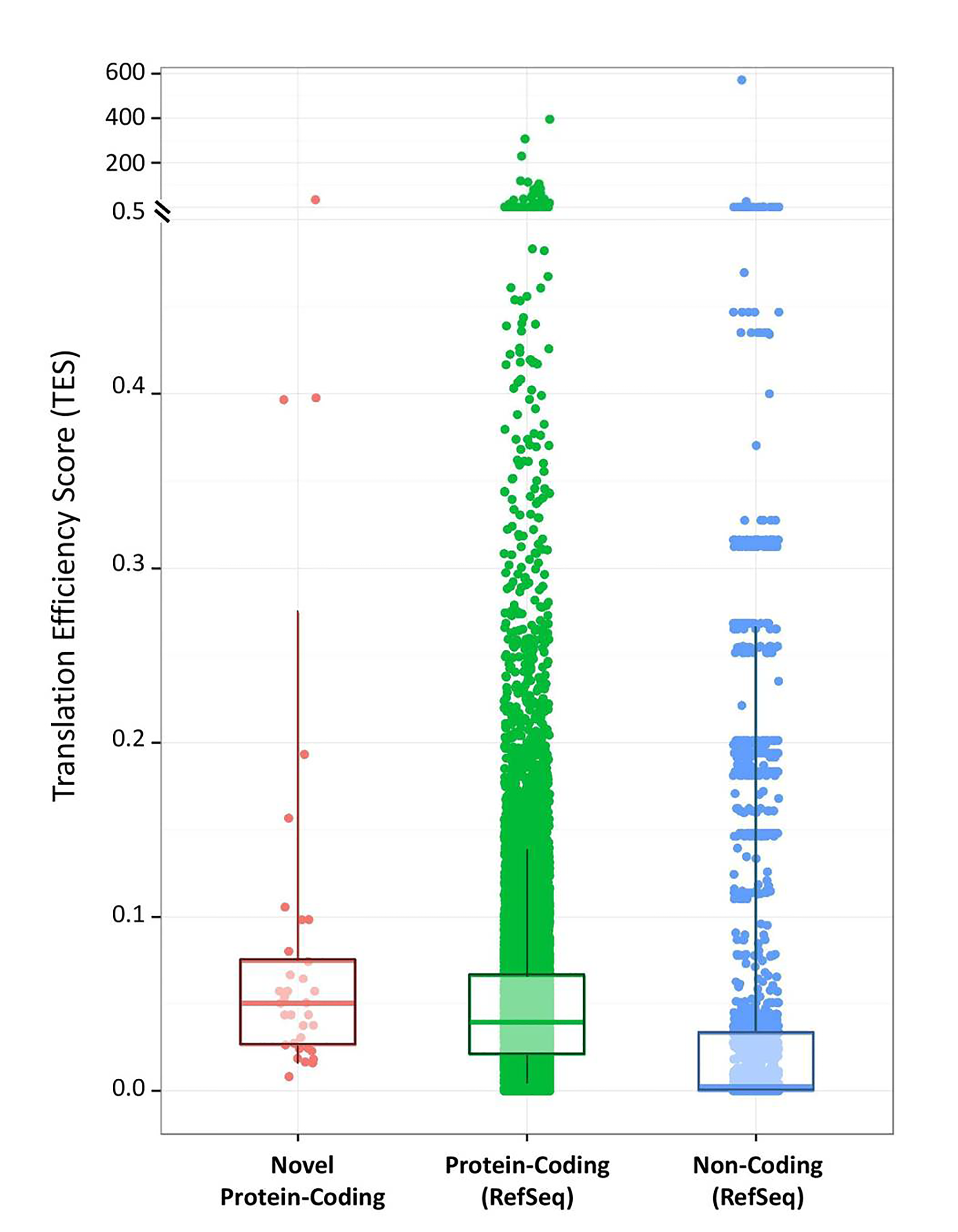

Supplement: S4 Fig — The box plot depicts the distribution of Translation Efficiency Scores (TES) across putative novel protein coding transcripts (34) identified in this study and RefSeq genes (coding and non coding). Centre lines show the median; box limits indicate the 25th and 75th percentiles; whiskers extend till the 5th and 95th percentiles. (TIF) [file pone.0147823.s004.tif]

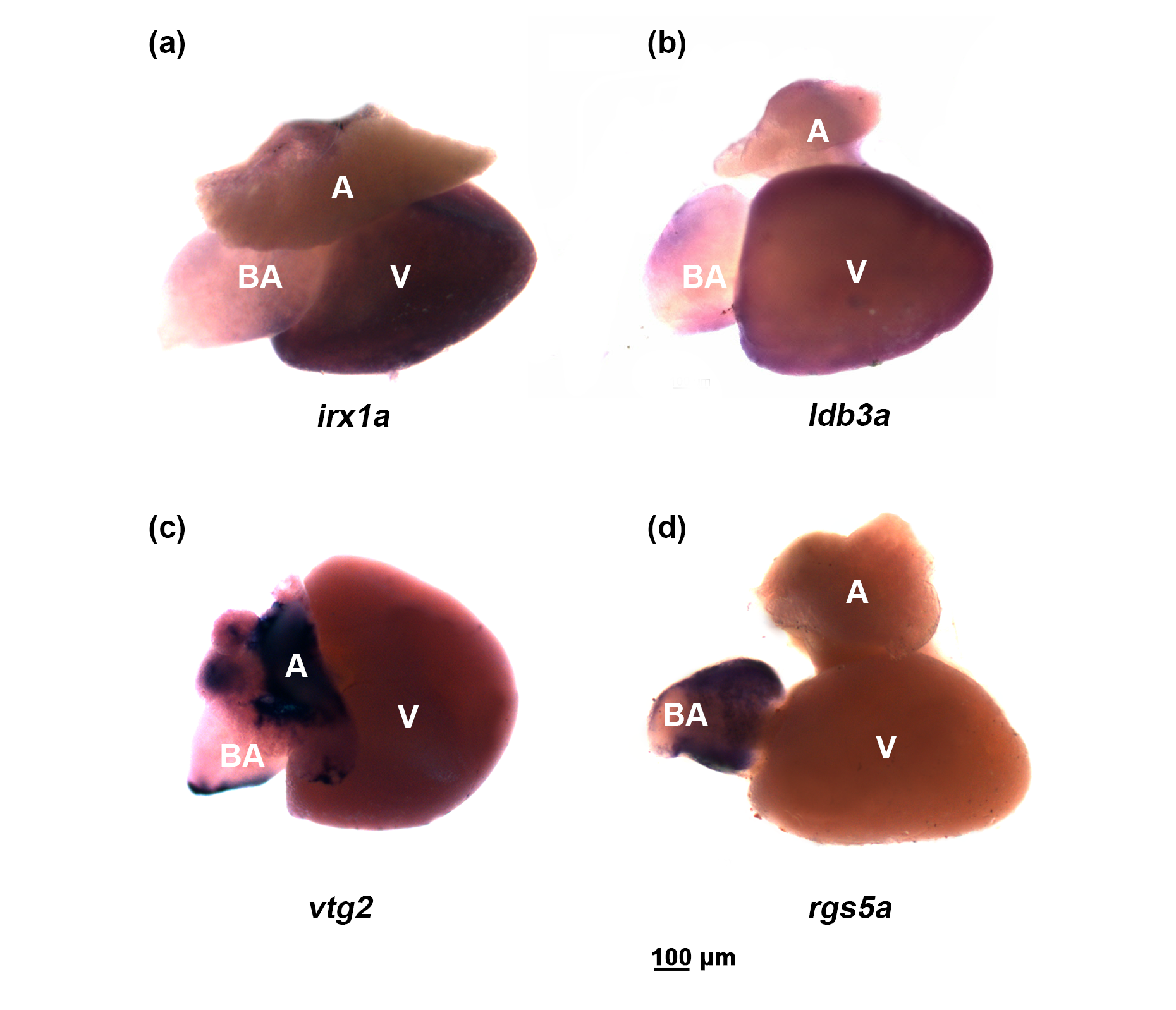

Supplement: S5 Fig — Ventricle-restricted genes (a) irx1a and (b) ldb3a, Atrium- restricted gene (c) vtg2, Bulbus arteriosus-restricted gene (d) rgs5a. (A) Atrium, (V) Ventricle and (BA) Bulbus arteriosus. Scale bar: 100 μm. See text for detailed information. (TIF) [file pone.0147823.s005.tif]
