# Supplementary material for: Chamber Specific Gene Expression Landscape of the Zebrafish Heart
Source: PLoS One. 2016 Jan 27;11(1):e0147823. doi: 10.1371/journal.pone.0147823 (PMC4729522; doi:10.1371/journal.pone.0147823)
Supplement: S1 Table — (DOCX) [file pone.0147823.s006.docx]

| **Primer Name** | **Forward Primer (5’-3’)** | **Reverse Primer (5’-3’)** |
| --- | --- | --- |
| myh6 | TGAAGACCTGAGAAGGCAAC | CAGTTCCTCGGTTCTCTGAA |
| vmhc | TCAGATGGCAGAGTTTGGAG | GCTTCCTTTACAGTTACAGTCTTTC |
| vmhcl | GCGATGCTGAAATGTCTGTT | CAGTCACAGTCTTGCCTCCT |
| tnnc1 | AGTTCCGTGCTGCGTTCGACATCTTCGTGC | CAGCTCATCCAAGTCAATGTAACCATCTCC |
| rgs5a | CAGAAGCCAGAGAATTCCATCGACCTTATT | CACGTCTTTGGTGAAGTGGTCGATATTCAC |
| elnb | CACAGTTTGGGAGTGGAGAGGGTTATTTAC | ACCAAGACCAGTACCTAGACCTAATCCTG |
| vtg1 | GATCTGAGCCACTGCCAGGAGAGAATCATG | TGAAACCAAGTGCTTCAGGACCTCGACAAT |
| ldb3a | TTCTACCAGCATTGCACCTG | ACCTACAGGGATCTGGATGG |
| hspb11 | AGCTCAACAGCAAACCCGAGAAATCCACTG | TCTTCTCCGTCTTTCCGCTCACCCTCAGCT |
| ldb3a* | TTCTACCAGCATTGCACCTG | ACCTACAGGGATCTGGATGG |
| irx1a* | CAGCTGGGCTACCCGCAGTATTTAAGTG | GCTCTCCAGATCTATCTCCTCTTCGTCC |
| vtg2* | CACAAAGATTAGCAATGCACCAGCCC | GCTGTCCACTGGATGGTCTCCAAATC |
| rgs5a* | TGGGAACTCTGCTTCAGAAGCCAGAG | TCTTTGGGCCCACCGGTCTGTATGAAGT |
| Novel1 | CTCAGACGGTGGAGAAGATGT | ATGCTTAGCCGTCGGACAACA |
| Novel 2 | TGCTTACATCCTCATGGTGCTCT | CAGCACCAAGATTATGATTCA |
| Novel 3 | TGATGGACAGAGACGTGGAAAC | TAGCCATGTTACATACGACTTG |

**S1 Table. List of genes primers.** Primer Sequences for the genes marked in asterisks (*) were used for probe preparation for whole mount *in situ* hybridization.
