# Supplementary material for: Chamber Specific Gene Expression Landscape of the Zebrafish Heart
Source: PLoS One. 2016 Jan 27;11(1):e0147823. doi: 10.1371/journal.pone.0147823 (PMC4729522; doi:10.1371/journal.pone.0147823)
Supplement: S2 Table — (DOCX) [file pone.0147823.s007.docx]

**S2 Table. List of genes differentially expressed in the zebrafish cardiac chambers and details on zebrafish mutants and human disease gene orthologs.**

|  | | |  | | | | **Expression (FPKM)** | | | | | | | **Mutant Phenotype** | | **Human Orthologs** | | **Diseases** | | |
| --- | --- | --- | --- | --- | --- | --- | --- | --- | --- | --- | --- | --- | --- | --- | --- | --- | --- | --- | --- | --- |
| **Gene_ID** | | | **Gene Name** | | | | **Atrium** | | | | **Ventricle** | | **BA** |  |  |  |  |  |  |  |
| **Atrium-enriched** | | | | | | | | | | | | | | | | | | | | |
| NM_001002085 | | tnnc1b | | | | 1236.49 | | 43.9386 | | | | 10.1804 | | NA | | TNNC1 | | | Cardiac | |
| NM_001005403 | | ba2 | | | | 3525.23 | | 315.092 | | | | 268.547 | | NA | | - | | | - | |
| NM_001013321 | | smtnl1 | | | | 386.066 | | 23.9325 | | | | 7.36551 | | NA | | SMTNL1 | | | - | |
| NM_001017822 | | zgc:110204 | | | | 14.4217 | | 0.680028 | | | | 1.59645 | | NA | | - | | | - | |
| NM_001025189 | | vtg5 | | | | 742.616 | | 63.7946 | | | | 70.5162 | | NA | | - | | | - | |
| NM_001039630 | | col18a1 | | | | 63.0337 | | 4.61528 | | | | 1.84916 | | Non-cardiac | | COL18A1 | | | Non-cardiac | |
| NM_001040333 | | zgc:136272 | | | | 28.5003 | | 2.50071 | | | | 2.74619 | | NA | | - | | | - | |
| NM_001044897 | | vtg1 | | | | 9067.64 | | 698.576 | | | | 829.197 | | NA | | - | | | - | |
| NM_001044913 | | vtg2 | | | | 711.321 | | 62.9353 | | | | 64.3738 | | NA | | - | | | - | |
| NM_001045294 | | vtg4 | | | | 503.414 | | 49.1318 | | | | 48.0344 | | NA | | - | | | - | |
| NM_001100137 | | mybphb | | | | 207.901 | | 15.5721 | | | | 1.97975 | | NA | | MYBPH | | | - | |
| NM_001110384 | | vtg2 | | | | 181.906 | | 4.06169 | | | | 9.18842 | | NA | | - | | | - | |
| NM_001111208 | | zgc:174259 | | | | 8.90383 | | 0.48427 | | | | 0.11036 | | NA | | - | | | - | |
| NM_001114553 | | vip | | | | 10.5193 | | 0.388707 | | | | 0.758428 | | NA | | VIP | | | - | |
| NM_001122610 | | vtg6 | | | | 155.076 | | 14.2338 | | | | 14.3837 | | NA | | - | | | - | |
| NM_001291886 | | apoc1l | | | | 41.0548 | | 0.00613722 | | | | 0.0268298 | | Non-cardiac | | - | | | - | |
| NM_198823 | | myh6 | | | | 6602.99 | | 249.489 | | | | 23.622 | | Cardiac | | MYH6 | | | Cardiac | |
| NM_201196 | | shox2 | | | | 14.3673 | | 0.248707 | | | | 1.20694 | | Cardiac | | SHOX2 | | | - | |
| NM_205628 | | dmrt1 | | | | 8.12039 | | 0.709061 | | | | 0.787386 | | NA | | DMRT1 | | | - | |
| NM_213305 | | scamp2 | | | | 9.03812 | | 0.00213549 | | | | 0.410108 | | NA | | SCAMP2 | | | - | |
| **Ventricle-enriched** | | | | | | | | | | | | | | | | | | | | |
| **Gene_ID** | **Gene name** | | | | **Expression (FPKM)** | | | | | | | | | | **Mutant phenotype** | **Human orthologs** | | | **Diseases** | |
|  |  |  |  |  | **Atrium** | | | | | **Ventricle** | | **Bulbus arteriosus** | | |  |  |  |  |  |  |
| NM_001005400 | Cthl | | | | 3.4129 | | | | | 93.2446 | | 2.71951 | | | NA | - | | | - | |
| NM_001005998 | zgc:101560 | | | | 4.34423 | | | | | 847.852 | | 26.5592 | | | NA | - | | | - | |
| NM_001017750 | acta1a | | | | 50.7138 | | | | | 914.134 | | 40.5735 | | | NA | ACTA1 | | | Non-cardiac | |
| NM_001039808 | slc2a1a | | | | 2.89443 | | | | | 63.9541 | | 1.96272 | | | Non-cardiac | SLC2A1 | | | Non-cardiac | |
| NM_001044765 | gpr22a | | | | 12.6735 | | | | | 586.269 | | 11.5635 | | | NA | GPR22 | | | - | |
| NM_001044775 | smarca2 | | | | 0.0915508 | | | | | 14.2944 | | 0.0912754 | | | NA | SMARCA2 | | | Non-cardiac | |
| NM_001044919 | tmcc2 | | | | 3.98761 | | | | | 46.0464 | | 2.87545 | | | NA | TMCC2 | | | - | |
| NM_001045309 | nrap | | | | 4.88512 | | | | | 49.9332 | | 3.56938 | | | NA | NRAP | | | - | |
| NM_001077148 | asb10 | | | | 1.6523 | | | | | 31.1109 | | 1.40725 | | | NA | ASB10 | | | Non-cardiac | |
| NM_001077463 | zgc:153665 | | | | 2.74423 | | | | | 28.0745 | | 0.845758 | | | NA | - | | | - | |
| NM_001077464 | vmhcl | | | | 47.153 | | | | | 14144.7 | | 302.654 | | | NA | - | | | - | |
| NM_001099427 | hspb11 | | | | 213.185 | | | | | 4974.54 | | 195.085 | | | NA | - | | | - | |
| NM_001100958 | hspb6 | | | | 9.55029 | | | | | 97.999 | | 7.19966 | | | NA | HSPB6 | | | - | |
| NM_001112733 | vmhc | | | | 2.1056 | | | | | 167.704 | | 4.15654 | | | Cardiac | MYH7 | | | Cardiac | |
| NM_001128760 | gabrr3a | | | | 1.86533 | | | | | 19.4035 | | 0.635893 | | | NA | GABRR3 | | | - | |
| NM_001145239 | scpp7 | | | | 0.466196 | | | | | 7.25877 | | 0.1336 | | | NA | - | | | - | |
| NM_001167855 | ndufa1 | | | | 3.00273 | | | | | 40.0544 | | 0.000345199 | | | NA | NDUFA1 | | | Non-cardiac | |
| NM_001199740 | tmem82 | | | | 2.52308 | | | | | 65.975 | | 2.52998 | | | NA | TMEM82 | | | - | |
| NM_001256639 | sctr | | | | 2.41395 | | | | | 47.6305 | | 0.632996 | | | NA | - | | | - | |
| NM_001271839 | aatf | | | | 0.0238857 | | | | | 6.79087 | | 0.00311205 | | | NA | AATF | | | - | |
| NM_001308608 | ckmt2a | | | | 13.8625 | | | | | 570.944 | | 11.9958 | | | NA | CKMT2 | | | - | |
| NM_131291 | fgf3 | | | | 0.594508 | | | | | 54.7187 | | 3.39998 | | | Non-cardiac | FGF3 | | | Non-cardiac | |
| NM_131806 | efnb3b | | | | 0.436315 | | | | | 17.9433 | | 0.994828 | | | NA | EFNB3 | | | - | |
| NM_152961 | fabp3 | | | | 162.506 | | | | | 1754.73 | | 66.7555 | | | Cardiac | FABP3 | | | - | |
| NM_198914 | sult2st1 | | | | 2.92917 | | | | | 36.686 | | 1.78923 | | | NA | - | | | - | |
| NM_200042 | mpped2 | | | | 4.23536 | | | | | 81.1859 | | 3.88955 | | | Cardiac | MPPED2 | | | - | |
| NM_200899 | myoz2a | | | | 2.63406 | | | | | 35.4099 | | 1.82965 | | | NA | MYOZ2 | | | Cardiac | |
| NM_201057 | irx2a | | | | 2.08381 | | | | | 86.6336 | | 4.55712 | | | Non-cardiac | IRX2 | | | - | |
| NM_207184 | irx1a | | | | 0.000277436 | | | | | 90.7505 | | 3.18654 | | | Cardiac | IRX1 | | | - | |
| NM_207638 | adra2b | | | | 3.83313 | | | | | 53.2256 | | 3.84826 | | | NA | ADRA2B | | | Non-cardiac | |
| NM_212708 | slc16a3 | | | | 36.0758 | | | | | 369.734 | | 12.2653 | | | NA | SLC16A3 | | | - | |
| NM_213361 | zgc:56382 | | | | 0.000751718 | | | | | 6.31591 | | 0.000182996 | | | NA | - | | | - | |
| **Bulbus arteriosus enriched genes** | | | | | | | | | | | | | | | | | | | | |
| **Gene_ID** | **Gene name** | | | **Expression (FPKM)** | | | | | | | | | | | **Mutant Phenotype** | | **Human Orthologs** | | | **Disease** |
|  |  |  |  | **Atrium** | | | | | **Ventricle** | | | **Bulbus arteriosus** | | |  |  |  |  |  |  |
| NM_001002197 | sh3bgrl2 | | | 3.61409 | | | | | 2.41356 | | | 73.4622 | | | NA | | SH3BGRL2 | | | - |
| NM_001002212 | prss35 | | | 6.66001 | | | | | 2.3426 | | | 97.072 | | | NA | | PRSS35 | | | - |
| NM_001002219 | htra1a | | | 3.70942 | | | | | 0.443279 | | | 89.2714 | | | Cardiac | | HTRA1 | | | Non-cardiac |
| NM_001002350 | zgc:92162 | | | 1.07539 | | | | | 0.359177 | | | 44.9486 | | | NA | | - | | | - |
| NM_001004109 | quo | | | 3.51113 | | | | | 3.71978 | | | 42.2425 | | | NA | | KIAA1755 | | | - |
| NM_001004512 | si:busm1-57f23.1 | | | 4.14944 | | | | | 6.91373 | | | 167.99 | | | NA | | - | | | - |
| NM_001005596 | rbfox1 | | | 1.33729 | | | | | 1.82608 | | | 21.9348 | | | Non-cardiac | | RBFOX1 | | | - |
| NM_001005979 | fbln5 | | | 4.49149 | | | | | 4.39324 | | | 146.333 | | | NA | | FBLN5 | | | Non-cardiac |
| NM_001006079 | osr1 | | | 5.65483 | | | | | 2.68782 | | | 64.7224 | | | Cardiac | | OSR1 | | | - |
| NM_001007310 | cyp1d1 | | | 4.09039 | | | | | 3.25167 | | | 111.942 | | | NA | | - | | | - |
| NM_001024448 | myh11a | | | 16.2422 | | | | | 14.4778 | | | 616.838 | | | Non-cardiac | | MYH11 | | | Non-cardiac |
| NM_001030141 | si:dkey-14k9.2 | | | 4.32488 | | | | | 3.67896 | | | 48.4512 | | | NA | | KIAA1644 | | | - |
| NM_001030153 | kcnj8 | | | 1.67611 | | | | | 3.03178 | | | 40.4245 | | | NA | | KCNJ8 | | | - |
| NM_001030282 | trpc6a | | | 1.4673 | | | | | 1.5459 | | | 47.5657 | | | NA | | TRPC6 | | | Non-cardiac |
| NM_001045185 | edn2 | | | 3.8226 | | | | | 8.15031 | | | 107.917 | | | NA | | EDN2 | | | - |
| NM_001048064 | elnb | | | 5.63746 | | | | | 4.73389 | | | 530.135 | | | NA | | ELN | | | Non-cardiac |
| NM_001080081 | cracr2b | | | 2.94835 | | | | | 1.03231 | | | 82.2682 | | | NA | | CRACR2B | | | - |
| NM_001082925 | s1pr3a | | | 1.77617 | | | | | 1.68003 | | | 53.9737 | | | NA | | S1PR3 | | | - |
| NM_001083813 | bcan | | | 1.3101 | | | | | 0.221525 | | | 16.2916 | | | NA | | BCAN | | | - |
| NM_001089422 | zgc:162595 | | | 10.9131 | | | | | 23.0765 | | | 390.04 | | | NA | | - | | | - |
| NM_001099978 | fhl3b | | | 3.70728 | | | | | 4.01725 | | | 136.006 | | | NA | | FHL3 | | | Non-cardiac |
| NM_001113642 | lrrc3 | | | 0.939151 | | | | | 0.293942 | | | 9.95316 | | | NA | | LRRC3 | | | - |
| NM_001114911 | itga8 | | | 1.21104 | | | | | 0.547899 | | | 111.521 | | | NA | | ITGA8 | | | Non-cardiac |
| NM_001115107 | sc:d0220 | | | 0.0350031 | | | | | 0.0370154 | | | 5.05311 | | | NA | | - | | | - |
| NM_001123515 | nrip2 | | | 4.15147 | | | | | 2.42031 | | | 51.9385 | | | NA | | NRIP2 | | | - |
| NM_001128276 | zgc:172120 | | | 1.11286 | | | | | 1.94348 | | | 25.3042 | | | NA | | - | | | - |
| NM_001135631 | atp2b1b | | | 4.53509 | | | | | 2.32105 | | | 54.4268 | | | NA | | ATP2B1 | | | - |
| NM_001145765 | si:ch211-10a23.2 | | | 2.52709 | | | | | 0.457024 | | | 25.8433 | | | NA | | - | | | - |
| NM_001159372 | ctnna2 | | | 1.20589 | | | | | 0.54678 | | | 18.7058 | | | NA | | CTNNA2 | | | - |
| NM_001162531 | syt11b | | | 1.88887 | | | | | 0.921191 | | | 19.6574 | | | NA | | SYT11 | | | - |
| NM_001164030 | has1 | | | 3.37773 | | | | | 1.28064 | | | 51.4568 | | | NA | | HAS1 | | | - |
| NM_131436 | npy8br | | | 1.6354 | | | | | 0.052479 | | | 21.128 | | | NA | | - | | | - |
| NM_131667 | gch2 | | | 1.09439 | | | | | 0.732158 | | | 26.7451 | | | NA | | GCH1 | | | Non-cardiac |
| NM_131691 | s1pr1 | | | 6.08869 | | | | | 4.06709 | | | 64.7211 | | | Cardiac | | S1PR1 | | | - |
| NM_173220 | has3 | | | 2.6742 | | | | | 0.609692 | | | 27.2364 | | | NA | | HAS3 | | | - |
| NM_199675 | tpm4b | | | 3.41481 | | | | | 2.58929 | | | 129.791 | | | NA | | TPM4 | | | - |
| NM_199962 | rgs5a | | | 9.19397 | | | | | 16.0921 | | | 1522.06 | | | NA | | RGS5 | | | Non-cardiac |
| NM_200177 | slmapb | | | 14.6992 | | | | | 12.9702 | | | 187.544 | | | NA | | SLMAP | | | - |
| NM_200723 | hpca | | | 5.34794 | | | | | 6.66686 | | | 71.8391 | | | NA | | HPCA | | | Non-cardiac |
| NM_200934 | tpm1 | | | 2.02483 | | | | | 8.60013 | | | 581.347 | | | NA | | TPM1 | | | Cardiac |
| NM_207089 | lox | | | 1.04186 | | | | | 1.6847 | | | 46.5373 | | | Cardiac | | LOX | | | - |
| NM_212620 | acta2 | | | 222.563 | | | | | 88.7679 | | | 3352.54 | | | Non-cardiac | | ACTA2 | | | Non-cardiac |
| NM_213549 | matn4 | | | 6.97297 | | | | | 10.9986 | | | 152.278 | | | NA | | MATN4 | | | - |
| NM_214688 | vcanb | | | 5.79195 | | | | | 7.30041 | | | 108.71 | | | NA | | VCAN | | | Non-cardiac |
