# Supplementary material for: Chamber Specific Gene Expression Landscape of the Zebrafish Heart
Source: PLoS One. 2016 Jan 27;11(1):e0147823. doi: 10.1371/journal.pone.0147823 (PMC4729522; doi:10.1371/journal.pone.0147823)
Supplement: S3 Table — (DOCX) [file pone.0147823.s008.docx]

**S3 Table. List of all putative novel protein-coding gene loci identified in the present study**

| **Gene_ID** | **Locus** | **Atrium** | **Ventricle** | **BA** | **Heart** |
| --- | --- | --- | --- | --- | --- |
| XLOC_170573 | chr25:11647112-12043697 | 4.83706 | 174.35 | 7.20942 | 11.2676 |
| XLOC_030968 | chr11:13811616-13824891 | 6.38243 | 170.023 | 61.8854 | 31.4674 |
| XLOC_042994 | chr12:47891305-47917357 | 2.35887 | 96.0988 | 13.7694 | 8.80802 |
| XLOC_152062 | chr23:1205560-1364126 | 2.56769 | 91.7545 | 8.15002 | 10.3257 |
| XLOC_215980 | chr6:15961436-16331582 | 3.71273 | 58.216 | 4.76025 | 6.19549 |
| XLOC_027405 | chr10:22111843-22149987 | 2.54964 | 50.3575 | 3.90252 | 7.09631 |
| XLOC_136909 | chr20:53192491-53337503 | 1.35691 | 38.5631 | 1.54649 | 3.75037 |
| XLOC_227537 | chr7:74552679-74627594 | 1.36588 | 35.896 | 3.45391 | 3.3872 |
| XLOC_043356 | chr12:1101462-1114820 | 1.00021 | 30.255 | 1.35757 | 2.36427 |
| XLOC_234792 | chr7:73964384-74006655 | 1.29586 | 29.3771 | 3.29295 | 2.95822 |
| XLOC_091989 | chr17:52045165-52141587 | 0.83177 | 29.339 | 3.75574 | 2.40581 |
| XLOC_159758 | chr23:35246205-35399695 | 1.3854 | 27.2876 | 1.71465 | 2.89392 |
| XLOC_208215 | chr5:71303755-71357849 | 1.71338 | 26.6306 | 2.88541 | 3.95169 |
| XLOC_181221 | chr3:57318375-57523546 | 2.12046 | 26.161 | 3.59946 | 5.20712 |
| XLOC_021007 | chr1:54677546-54749048 | 1.26427 | 24.9029 | 1.48686 | 2.9132 |
| XLOC_160341 | chr23:43025215-43045979 | 1.03855 | 22.8296 | 1.49243 | 2.20466 |
| XLOC_006309 | Zv9_scaffold3459:298822-404403 | 1.30974 | 21.405 | 1.78176 | 3.73421 |
| XLOC_100534 | chr18:30100351-30363305 | 1.5129 | 20.1874 | 2.23452 | 2.47044 |
| XLOC_038320 | chr11:43283232-43398608 | 1.1492 | 19.6634 | 2.44804 | 1.73315 |
| XLOC_141465 | chr21:8124180-8192601 | 0.45266 | 17.5972 | 0.54177 | 1.22788 |
| XLOC_102595 | chr18:7101-12638 | 0.63244 | 14.388 | 0.79906 | 1.64738 |
| XLOC_207391 | chr5:60438065-60530219 | 0.35961 | 12.1436 | 0.93756 | 0.97801 |
| XLOC_220636 | chr7:6807948-6810255 | 0.63656 | 10.3666 | 0.9539 | 2.18114 |
