# Supplementary material for: Chamber Specific Gene Expression Landscape of the Zebrafish Heart
Source: PLoS One. 2016 Jan 27;11(1):e0147823. doi: 10.1371/journal.pone.0147823 (PMC4729522; doi:10.1371/journal.pone.0147823)
Supplement: S4 Table — (DOCX) [file pone.0147823.s009.docx]

**S4 Table. KEGG pathway analysis**

| **PathwayName** | **#Gene** | **Enrichment Ratio** | **P-value (Raw)** | **P-value (Adjusted)** |
| --- | --- | --- | --- | --- |
|  |  |  |  |  |
| **Atrium – Upregulated gene pathways** | | | | |
| Metabolic pathways | 47 | 2.63 | 2.21E-009 | 1.41E-007 |
| PPAR signaling pathway | 7 | 6.98 | 6.34E-005 | 0.0015 |
| Tryptophan metabolism | 6 | 8.48 | 7.01E-005 | 0.0015 |
| Starch and sucrose metabolism | 5 | 8.68 | 0.0003 | 0.0032 |
| Steroid hormone biosynthesis | 5 | 8.21 | 0.0003 | 0.0032 |
| Drug metabolism - other enzymes | 5 | 8.68 | 0.0003 | 0.0032 |
| Neuroactive ligand-receptor interaction | 17 | 2.59 | 0.0004 | 0.0037 |
| Glycolysis / Gluconeogenesis | 6 | 5.79 | 0.0006 | 0.0048 |
| Histidine metabolism | 4 | 9.73 | 0.0007 | 0.005 |
| Phenylalanine metabolism | 3 | 13.03 | 0.0014 | 0.0081 |
| Primary bile acid biosynthesis | 3 | 13.03 | 0.0014 | 0.0081 |
| Porphyrin and chlorophyll metabolism | 4 | 7.37 | 0.002 | 0.0085 |
| Pentose phosphate pathway | 4 | 7.84 | 0.0016 | 0.0085 |
| Glycine, serine and threonine metabolism | 4 | 7.6 | 0.0018 | 0.0085 |
| Metabolism of xenobiotics by cytochrome P450 | 4 | 7.37 | 0.002 | 0.0085 |
| Other types of O-glycan biosynthesis | 4 | 6.75 | 0.0028 | 0.0112 |
| Phenylalanine, tyrosine and tryptophan biosynthesis | 2 | 20.26 | 0.0039 | 0.0147 |
| Ubiquinone and other terpenoid-quinone biosynthesis | 2 | 15.2 | 0.0071 | 0.0252 |
| Drug metabolism - cytochrome P450 | 3 | 5.88 | 0.0141 | 0.0392 |
| Tyrosine metabolism | 3 | 5.88 | 0.0141 | 0.0392 |
| Retinol metabolism | 3 | 6.08 | 0.0129 | 0.0392 |
| Pantothenate and CoA biosynthesis | 2 | 11.05 | 0.0135 | 0.0392 |
| alpha-Linolenic acid metabolism | 2 | 11.05 | 0.0135 | 0.0392 |
| **Atrium – Downregulated gene pathways** | | | | |
| Focal adhesion | 8 | 6.6 | 3.22E-005 | 0.0006 |
| Cell adhesion molecules (CAMs) | 5 | 8.6 | 0.0003 | 0.003 |
| MAPK signaling pathway | 7 | 4.35 | 0.0012 | 0.008 |
| Tight junction | 5 | 5.87 | 0.0017 | 0.0085 |
| Melanogenesis | 4 | 5.83 | 0.0051 | 0.0166 |
| Vascular smooth muscle contraction | 4 | 6.07 | 0.0044 | 0.0166 |
| Cytokine-cytokine receptor interaction | 4 | 5.6 | 0.0058 | 0.0166 |
| Dorso-ventral axis formation | 2 | 13.9 | 0.0091 | 0.0222 |
| Cardiac muscle contraction | 3 | 6.77 | 0.01 | 0.0222 |
| Regulation of actin cytoskeleton | 5 | 3.75 | 0.0112 | 0.0224 |
| Wntsignaling pathway | 4 | 4.06 | 0.0174 | 0.0316 |
| Gap junction | 3 | 4.63 | 0.0274 | 0.0457 |
| **Ventricle – Upregulated gene pathways** | | | | |
| Metabolic pathways | 214 | 5.24 | 1.48E-093 | 1.55E-091 |
| Oxidative phosphorylation | 76 | 14.65 | 6.38E-071 | 3.35E-069 |
| Valine, leucine and isoleucine degradation | 33 | 19.08 | 3.95E-037 | 1.38E-035 |
| Citrate cycle (TCA cycle) | 22 | 17.73 | 4.86E-024 | 1.28E-022 |
| Propanoatemetabo-lism | 22 | 17.21 | 1.33E-023 | 2.79E-022 |
| Glycolysis / Gluconeogenesis | 28 | 11.82 | 1.66E-023 | 2.91E-022 |
| Cardiac muscle contraction | 30 | 9.98 | 1.83E-022 | 2.74E-021 |
| Fatty acid metabolism | 22 | 15.4 | 4.66E-022 | 6.12E-021 |
| Pyruvate metabolism | 19 | 13.3 | 1.32E-017 | 1.54E-016 |
| Lysine degradation | 17 | 8.53 | 4.81E-012 | 5.05E-011 |
| beta-Alanine metabolism | 12 | 13.3 | 1.34E-011 | 1.28E-010 |
| Tryptophan metabolism | 15 | 9.28 | 2.19E-011 | 1.92E-010 |
| Glyoxylate and dicarboxylate metabolism | 10 | 14.78 | 1.81E-010 | 1.46E-009 |
| Butanoate metabolism | 11 | 10.84 | 1.52E-009 | 1.14E-008 |
| Arginine and proline metabolism | 14 | 5.82 | 8.62E-008 | 6.03E-007 |
| Porphyrin and chlorophyll metabolism | 10 | 8.06 | 2.28E-007 | 1.50E-006 |
| Fructose and mannose metabolism | 11 | 6.81 | 3.85E-007 | 2.25E-006 |
| Peroxisome | 15 | 4.87 | 3.64E-007 | 2.25E-006 |
| Pentose and glucuronate interconversions | 8 | 9.25 | 1.15E-006 | 6.36E-006 |
| Ubiquinone and other terpenoid-quinone biosynthesis | 5 | 16.63 | 3.79E-006 | 1.99E-005 |
| Starch and sucrose metabolism | 9 | 6.84 | 4.25E-006 | 2.13E-005 |
| Fatty acid elongation in mitochondria | 6 | 11.4 | 6.46E-006 | 3.08E-005 |
| Pentose phosphate pathway | 8 | 6.86 | 1.42E-005 | 6.48E-005 |
| Limonene and pinene degradation | 4 | 17.73 | 2.81E-005 | 0.0001 |
| Alanine, aspartate and glutamate metabolism | 8 | 6.08 | 3.71E-005 | 0.0002 |
| PPAR signaling pathway | 10 | 4.36 | 8.55E-005 | 0.0003 |
| Cysteine and methionine metabolism | 8 | 5.32 | 0.0001 | 0.0004 |
| Ascorbate and aldarate metabolism | 5 | 7.82 | 0.0003 | 0.0011 |
| Ribosome | 11 | 3.29 | 0.0005 | 0.0018 |
| Focal adhesion | 19 | 2.31 | 0.0007 | 0.0024 |
| Phenylalanine, tyrosine and tryptophan biosynthesis | 3 | 13.3 | 0.001 | 0.0032 |
| Glycerolipid metabolism | 7 | 4.33 | 0.001 | 0.0032 |
| Proteasome | 8 | 3.87 | 0.001 | 0.0032 |
| Glycine, serine and threonine metabolism | 6 | 4.99 | 0.0011 | 0.0034 |
| Metabolism of xenobiotics by cytochrome P450 | 6 | 4.84 | 0.0013 | 0.0039 |
| Glutathione metabolism | 7 | 4.14 | 0.0014 | 0.0041 |
| RNA degradation | 9 | 3.28 | 0.0016 | 0.0045 |
| Purine metabolism | 15 | 2.39 | 0.0017 | 0.0047 |
| Amino sugar and nucleotide sugar metabolism | 7 | 3.8 | 0.0023 | 0.0062 |
| Lysine biosynthesis | 2 | 17.73 | 0.0041 | 0.0108 |
| Retinol metabolism | 5 | 4.43 | 0.0048 | 0.0123 |
| Biosynthesis of unsaturated fatty acids | 4 | 5.6 | 0.0049 | 0.0123 |
| Aminoacyl-tRNA biosynthesis | 5 | 3.59 | 0.012 | 0.0286 |
| Steroid hormone biosynthesis | 5 | 3.59 | 0.012 | 0.0286 |
| Tight junction | 12 | 2.07 | 0.0137 | 0.0313 |
| Histidine metabolism | 4 | 4.26 | 0.0134 | 0.0313 |
| Phenylalanine metabolism | 3 | 5.7 | 0.0141 | 0.0315 |
| Galactose metabolism | 4 | 4.09 | 0.0154 | 0.0337 |
| Terpenoid backbone biosynthesis | 3 | 5.32 | 0.0172 | 0.0369 |
| Glycerophospholipid metabolism | 7 | 2.59 | 0.0184 | 0.0386 |
| Adipocytokinesignaling pathway | 7 | 2.55 | 0.0197 | 0.0406 |
| Inositol phosphate metabolism | 6 | 2.71 | 0.0231 | 0.0466 |
| Nucleotide excision repair | 5 | 3.02 | 0.024 | 0.0475 |
| Calcium signaling pathway | 15 | 1.76 | 0.0253 | 0.0492 |
| **Ventricle – Downregulated gene pathways** | | | | |
| Calcium signaling pathway | 20 | 2.99 | 1.45E-005 | 0.001 |
| Cell adhesion molecules (CAMs) | 12 | 3.88 | 6.25E-005 | 0.0022 |
| Cytokine-cytokine receptor interaction | 12 | 3.16 | 0.0004 | 0.0047 |
| Natural killer cell mediated cytotoxicity | 10 | 3.82 | 0.0003 | 0.0047 |
| NOD-like receptor signaling pathway | 7 | 5.17 | 0.0004 | 0.0047 |
| Gap junction | 12 | 3.49 | 0.0002 | 0.0047 |
| Phagosome | 12 | 2.76 | 0.0015 | 0.015 |
| Jak-STAT signaling pathway | 9 | 2.94 | 0.0036 | 0.0315 |
| **BA – Upregulated gene pathways** |  |  |  |  |
| Focal adhesion | 20 | 6.05 | 1.76E-010 | 8.10E-009 |
| MAPK signaling pathway | 17 | 3.87 | 2.55E-006 | 5.87E-005 |
| Cytokine-cytokine receptor interaction | 11 | 5.65 | 4.48E-006 | 6.87E-005 |
| Gap junction | 10 | 5.66 | 1.19E-005 | 0.0001 |
| Regulation of actin cytoskeleton | 14 | 3.85 | 2.06E-005 | 0.0002 |
| Hedgehog signaling pathway | 7 | 7.47 | 4.13E-005 | 0.0003 |
| ECM-receptor interaction | 7 | 7.13 | 5.62E-005 | 0.0004 |
| Vascular smooth muscle contraction | 9 | 5.01 | 8.47E-005 | 0.0004 |
| TGF-beta signaling pathway | 8 | 5.88 | 6.71E-005 | 0.0004 |
| Notch signaling pathway | 6 | 7.79 | 0.0001 | 0.0005 |
| Cardiac muscle contraction | 7 | 5.79 | 0.0002 | 0.0008 |
| GnRHsignaling pathway | 8 | 4.65 | 0.0003 | 0.0011 |
| Purine metabolism | 9 | 3.57 | 0.001 | 0.0035 |
| Wntsignaling pathway | 9 | 3.35 | 0.0016 | 0.0053 |
| Melanogenesis | 7 | 3.74 | 0.0028 | 0.0086 |
| Adherens junction | 6 | 4.23 | 0.0031 | 0.0087 |
| Nitrogen metabolism | 3 | 9.93 | 0.0032 | 0.0087 |
| Endocytosis | 10 | 2.56 | 0.0064 | 0.0164 |
| Dorso-ventral axis formation | 3 | 7.64 | 0.0069 | 0.0167 |
| Intestinal immune network for IgA production | 3 | 6.85 | 0.0094 | 0.0216 |
| Cell adhesion molecules (CAMs) | 5 | 3.15 | 0.0217 | 0.0475 |
| **BA – Downregulated gene pathways** | | | | |
| Metabolic pathways | 158 | 6.44 | 9.00E-082 | 6.48E-080 |
| Oxidative phosphorylation | 66 | 21.18 | 5.26E-071 | 1.89E-069 |
| Cardiac muscle contraction | 30 | 16.61 | 6.92E-029 | 1.66E-027 |
| Valine, leucine and isoleucine degradation | 24 | 23.11 | 1.05E-027 | 1.89E-026 |
| Glycolysis / Gluconeogenesis | 21 | 14.76 | 2.29E-019 | 3.30E-018 |
| Propanoate metabolism | 16 | 20.84 | 5.86E-018 | 7.03E-017 |
| Fatty acid metabolism | 16 | 18.65 | 5.43E-017 | 5.59E-016 |
| Citrate cycle (TCA cycle) | 15 | 20.13 | 1.25E-016 | 1.13E-015 |
| Pyruvate metabolism | 15 | 17.48 | 1.68E-015 | 1.34E-014 |
| Glyoxylate and dicarboxylate metabolism | 10 | 24.6 | 1.20E-012 | 8.64E-012 |
| Calcium signaling pathway | 24 | 4.68 | 4.60E-010 | 3.01E-009 |
| Butanoate metabolism | 9 | 14.76 | 4.73E-009 | 2.84E-008 |
| Fatty acid elongation in mitochondria | 7 | 22.14 | 8.69E-009 | 4.81E-008 |
| beta-Alanine metabolism | 8 | 14.76 | 3.47E-008 | 1.78E-007 |
| Arginine and proline metabolism | 10 | 6.92 | 1.63E-006 | 7.82E-006 |
| Lysine degradation | 9 | 7.52 | 2.65E-006 | 1.19E-005 |
| Starch and sucrose metabolism | 7 | 8.86 | 1.13E-005 | 4.79E-005 |
| Cysteine and methionine metabolism | 7 | 7.75 | 2.83E-005 | 0.0001 |
| Tryptophan metabolism | 7 | 7.21 | 4.62E-005 | 0.0002 |
| Fructose and mannose metabolism | 7 | 7.21 | 4.62E-005 | 0.0002 |
| Alanine, aspartate and glutamate metabolism | 6 | 7.59 | 0.0001 | 0.0003 |
| Phenylalanine, tyrosine and tryptophan biosynthesis | 3 | 22.14 | 0.0002 | 0.0006 |
| Phenylalanine metabolism | 4 | 12.65 | 0.0002 | 0.0006 |
| Peroxisome | 8 | 4.32 | 0.0005 | 0.0015 |
| Tyrosine metabolism | 5 | 7.14 | 0.0006 | 0.0017 |
| Biosynthesis of unsaturated fatty acids | 4 | 9.32 | 0.0008 | 0.0022 |
| PPAR signaling pathway | 6 | 4.36 | 0.0025 | 0.0067 |
| Glutathione metabolism | 5 | 4.92 | 0.0034 | 0.0087 |
| Purine metabolism | 10 | 2.65 | 0.0048 | 0.0119 |
| Pentose phosphate pathway | 4 | 5.71 | 0.005 | 0.012 |
| Glycine, serine and threonine metabolism | 4 | 5.54 | 0.0056 | 0.013 |
| Insulin signaling pathway | 9 | 2.54 | 0.0096 | 0.0216 |
| Inositol phosphate metabolism | 5 | 3.75 | 0.0106 | 0.0231 |
| Nicotinate and nicotinamide metabolism | 3 | 6.33 | 0.0113 | 0.0239 |
| Galactose metabolism | 3 | 5.11 | 0.0203 | 0.0418 |
| Regulation of actin cytoskeleton | 11 | 2.02 | 0.0218 | 0.0436 |
| Melanogenesis | 7 | 2.5 | 0.0227 | 0.0442 |
| Valine, leucine and isoleucine biosynthesis | 2 | 8.05 | 0.0245 | 0.0464 |
